# Comprehensive multiomics analysis of the signatures of gastric mucosal bacteria and plasma metabolites across different stomach microhabitats in the development of gastric cancer

**DOI:** 10.1007/s13402-024-00965-3

**Published:** 2024-07-04

**Authors:** Bingsen Wang, Jiahui Luan, Weidong Zhao, Junbao Yu, Anqing Li, Xinxin Li, Xiaoqin Zhong, Hongyun Cao, Ruicai Wang, Bo Liu, Shiyong Lu, Mei Shi

**Affiliations:** 1 State Key Laboratory of Microbial Technology, Shandong University, Qingdao, 266237 China; 2 Shandong University-Zibo Municipal Hospital Research Center of Human Microbiome and Health, Zibo, 255400 China; 3 Department of gastroenterology, Zibo Municipal Hospital, Zibo, 255400 China; 4 Department of Clinical Microbiology, Zibo City Key Laboratory of Respiratory Infection and Clinical Microbiology, Zibo City Engineering Technology Research Center of Etiology Molecular Diagnosis, Zibo Municipal Hospital, Zibo, 255400 China; 5 Department of Pulmonary and Critical Care Medicine, Shandong Institute of Respiratory Diseases, The First Affiliated Hospital of Shandong First Medical University, Shandong Provincial Qianfoshan Hospital, Shandong University, Jinan, 250014 China; 6 Department of Pathology, Zibo Municipal Hospital, Zibo, 255400 China

**Keywords:** Gastric microbiota, Plasma metabolites, Gastric cancer, *Helicobacter pylori*, Multi-omic analyses

## Abstract

**Purpose:**

As an important component of the microenvironment, the gastric microbiota and its metabolites are associated with tumour occurrence, progression, and metastasis. However, the relationship between the gastric microbiota and the development of gastric cancer is unclear. The present study investigated the role of the gastric mucosa microbiome and metabolites as aetiological factors in gastric carcinogenesis.

**Methods:**

Gastric biopsies from different stomach microhabitats (n = 70) were subjected to 16S rRNA gene sequencing, and blood samples (n = 95) were subjected to untargeted metabolome (gas chromatography‒mass spectrometry, GC‒MS) analyses. The datasets were analysed using various bioinformatics approaches.

**Results:**

The microbiota diversity and community composition markedly changed during gastric carcinogenesis. High *Helicobacter. pylori* colonization modified the overall diversity and composition of the microbiota associated with gastritis and cancer in the stomach. Most importantly, analysis of the functional features of the microbiota revealed that nitrate reductase genes were significantly enriched in the tumoral microbiota, while urease-producing genes were significantly enriched in the microbiota of *H. pylori*-positive patients. A panel of 81 metabolites was constructed to discriminate gastric cancer patients from gastritis patients, and a panel of 15 metabolites was constructed to discriminate *H. pylori-positive* *patients* from *H. pylori*-negative patients. receiver operator characteristic (ROC) curve analysis identified a series of gastric microbes and plasma metabolites as potential biomarkers of gastric cancer.

**Conclusion:**

The present study identified a series of signatures that may play important roles in gastric carcinogenesis and have the potential to be used as biomarkers for diagnosis and for the surveillance of gastric cancer patients with minimal invasiveness.

**Supplementary Information:**

The online version contains supplementary material available at 10.1007/s13402-024-00965-3.

## Introduction

Gastric cancer (GC) is one of the leading causes of cancer-related mortality worldwide, and its incidence is highest in Eastern Asia, emphasizing the need to elucidate the major components and functional molecules that play pivotal roles in GC pathogenesis and develop novel diagnostic markers and therapeutic targets [1, 2]. Before cancer becomes clinically apparent, a prolonged precancerous process progresses from superficial gastritis to atrophic gastritis, intestinal metaplasia, and dysplasia [3]. With respect to screening for GC, traditional invasive procedures rely on endoscopy and biopsy, which are largely ineffective and show conflicting results. GC is often diagnosed at an advanced stage, and it is difficult to obtain satisfactory treatment effects due to its asymptomatic and nonspecific symptoms in the early stage [4]. Therefore, it is crucial to identify noninvasive biomarkers associated with the occurrence and progression of GC for the detection of early-stage disease and improvement of GC prognosis.

As part of the tumour microenvironment, the gastric microbiota has attracted increasing attention because it contributes to GC initiation, progression, and metastasis [5]. Compared to studies related to the gut microbiota, however, the gastric microbiota has been relatively understudied. Recent studies have demonstrated that different stages of gastric carcinogenesis and surgical treatment can result in significant changes in gastric microbiota profiles [6, 7]. The microbial communities of the stomach exhibit complex ecosystems that are significantly different from those of the mouth and oesophagus, and they have distinct anatomical locations [8]. It is widely accepted that the stomach is the exclusive habitat for *H. pylori* and that it is an inhospitable environment for a number of commensal bacterial species due to the acidic conditions and the presence of bactericidal antibiotics [9, 10]. Although it has been suggested that *H. pylori* colonization induces dysbiosis of the gastric microbiota, the specific relationship between *H. pylori* and other gastric microbiota is not fully understood [3, 9–11]. For example, Sung et al. [12] showed that *H. pylori* infection leads to decreased gastric microbiota diversity, which is restored after *H. pylori* eradication. Although infection with *H. pylori* is a risk factor for gastric adenocarcinoma and is responsible for approximately 75% of global gastric cancers, the incidence of gastric cancer attributable to *H. pylori* is only 1–2%, indicating that the roles of biological factors other than *H. pylori* are less well defined [13, 14]. Moreover, studies have implied that the elimination of *H. pylori* does not effectively inhibit GC development. Thus, further research is required to explore the complex mechanism of GC carcinogenesis involving other bacterial species in the microbiota. At present, etiological studies of meta-cohorts have confirmed the role of bacteria other than *H. pylori* in gastric carcinogenesis. When *H. pylori* disappears in patients who progress through chronic gastritis, intestinal metaplasia, or dysplasia towards gastric carcinoma, other anaerobic or facultative anaerobic bacteria become predominant [15, 16]. When other bacteria colonize the stomach under weakly acidic conditions, they may continue to trigger gastric carcinogenesis through genotoxicity and inflammatory modulation [17–19]. Recent research has shed light on the involvement of probiotics and probiotics in GC-related inflammation in the treatment of *H. pylori* infection. However, there is limited evidence characterizing the prebiotic microbiota during gastric carcinogenesis [20, 21]. Because it is difficult to define the specific microbiota associated with the cause of gastric cancer and the beneficial effects resulting from *H. pylori* eradication, the extent to which the microbiota operates in the gut remains unclear.

Metabolomics provides new approaches for exploring a set of metabolites synthesized by complex physiological ecosystems, and it is an important omics technique for identifying the functional molecules contributing to a particular physiological and pathological stage. However, metabolomics has scarcely been used in GC research. An increasing number of studies have shown that metabolites in human fluid samples, including serum and urine, can be important downstream or typical clinical biomarkers in different diseases [22–25]. Significant alterations have been reported in several metabolites of serum and plasma associated with GC, including amino acids and their derivatives, represented by tryptophan, glutamic acid, and kynurenine (a tryptophan metabolite) [26–28]. Moreover, the differences in the distribution of *H. pylori* infection across the groups of patients do not lead to changes in the metabolomic profile of *H. pylori* [22]. To date, however, relatively few studies have verified metabolites that may be precise diagnostic indicators for gastric cancer [29].

In the present study, several superficial gastritis (SG) patients, atrophic gastritis (AG) patients, and GC volunteers without preoperative chemotherapy were enrolled, and mucosal tissues were collected from different stomach microhabitats. The present study analysed the diversity and composition of the gastric microbiota across inflammatory and precancerous microhabitats, as well as changes in host metabolism involved in microbiota dysbiosis. Major shifts in the gastric cancer microbiota relative to carcinogenesis were characterized, providing an accurate understanding of the roles of *Helicobacter* and other bacteria in GC occurrence and progression. The differences in plasma metabolites between GC patients and chronic gastritis patients were evaluated to identify sensitive plasma biomarkers for GC using LC‒MS/MS, which may help to identify novel diagnostic tools and potential therapeutic targets.

## Materials and methods

### Clinical sample collection

In total, 63 superficial gastritis, 126 atrophic gastritis, and 14 GC mucosa biopsy samples from 70 patients without preoperative chemotherapy were collected at Zibo Hospital (Zibo, China) from September 2018 to April 2020. Stomach mucosa was obtained from patients infected with *H. pylori* under pathological conditions of superficial gastritis (PSG), atrophic gastritis (PAG), and primary GC (PC), as well as from patients without *H. pylori* under pathological conditions of superficial gastritis (NSG), atrophic gastritis (NAG) and primary GC (NC). Gastric mucosa (horn, antrum, and body) biopsies and blood samples were obtained from each patient. The main diagnostic criteria for *H. pylori* positivity in samples were gastric mucosal tissue rapid urease test (RUT) and ^14^C-urea breath test (^14^C-UBT) results. The collected tumour and peritumoral (2–5 cm adjacent to tumour tissue) tissues were subjected to pathological diagnosis via endoscopic biopsy. All biopsy samples were screened for DNA concentration and quality, and qualified samples were used for 16S rRNA amplification and next-generation DNA sequencing. The subsequent bacterial taxonomic annotation results obtained by subsequent 16S rRNA amplicon sequencing were used to correct false-negative (or false-positives) samples in clinical diagnosis. Volunteers were excluded if they met any of the following criteria: body mass index (BMI) > 30 (BMI = weight in kilograms divided by the height in metres squared); used any type of antibiotic or oral Chinese patent medicine with antibacterial effect within one month; consumed food or health products containing probiotics, prebiotics, or synbiotics in the previous month; received preoperative chemotherapy, radiotherapy, or other biological treatment before endoscopic biopsy; used any other drugs that affect the gastrointestinal microbiota within the last month; treated with proton-pump inhibitors (PPI) or antibiotics within 1 month before endoscopy; diagnosed with cardiovascular, diabetes, or renal disorders; infected with human immunodeficiency virus or diagnosed with severe enterological diseases; or complicated with other malignancies within 5 years. Basic personal and clinical data regarding the individuals are provided in Supplementary Table 1. The present study was approved by the Ethics Committee of Zibo Municipal Hospital (20200221). Written informed consent was obtained from all of the participants.

### DNA extraction, amplicon library construction, and sequencing

Microbial genomic DNA was extracted from mucosal biopsy samples with an E.Z.N.A.^®^ DNA Kit (Omega Biotek, Norcross, GA, USA). The quality and concentration of the DNA were measured by 1.0% agarose gel electrophoresis and a NanoDrop® ND-2000 spectrophotometer (Thermo Scientific Inc., USA), and the DNA was stored at −80 °C. The V3-V4 region of the 16S rRNA gene was amplified by an ABI GeneAmp® 9700 PCR thermocycler (ABI, CA, USA). The following primer pairs were used: 338 F, 5’-ACTCCTACGGGAGGCAGCAG-3’; and 806 R, 5’-GGACTACHVGGGTWTCTAAT-3’. The PCR mixture included 10 ng of template DNA, 0.8 μL of each primer (5 μM), 0.4 μL of Fast Pfu polymerase, 2 μL of 2.5 mM dNTPs, 4 μL of 5 × Fast Pfu buffer, and ddH_2_O to a volume of 20 µL. The PCR products were extracted from 2% agarose gels, purified using an AxyPrep DNA Gel Extraction Kit (Axygen Biosciences, Union City, CA, USA), and quantified using a Quantus™ Fluorometer (Promega, USA).

The purified PCR products were loaded in equimolar amounts and paired-end sequenced on an Illumina MiSeq PE300 platform (Illumina, San Diego, USA).

### Data processing and statistical analysis

The raw FASTQ files were demultiplexed using an in-house R script, quality-filtered by fastp (0.19.6), and merged by FLASH (1.2.7) based on several parameters. First, the 300 bp reads were truncated at any site receiving an average quality score of <20 over a 50 bp sliding window, and the truncated reads shorter than 50 bp or containing ambiguous characters were discarded. Second, the read pairs with overlaps longer than 10 bp were assembled, with a maximum mismatch ratio of 0.2 for the overlap region. Reads that could not be assembled were discarded. Third, UPARSE 7.1 was used to cluster the processed sequences into operational taxonomic units (OTUs) at the 97% identity level. The sequence with the highest number in each OTU was regarded as the sequence of that OTU. Finally, to reduce the influence of sample density and sequencing depth, random rarefaction of sample reads, according to the smallest read length of 24,000, was performed for downstream analysis.

Statistical analysis and visualization software included R (v4.0.5), STAMP (v), GraphPad Prism (v6.0), and Metabanalyst (v5.0, http://www.metaboanalyst.ca). Linear discriminant analysis (LDA) effect size (LEfSe) was performed to characterize the bacterial taxa in the gastric microbiota (http://huttenhower.sph.harvard.edu/lefse/).

### Metabolite extraction and LC‒MS/MS analysis

A total of 95 plasma samples and 18 QC samples were filtered for LC‒MS/MS analysis. After thawing at 4 °C, 100 μL of sample was placed in a 96-well plate. Then, 300 μL of extract (methanol: ACN = 2:1, V: V, precooled at − 20 °C) and two internal standard buffers (10 μL of each buffer) were added, and the mixture was vortexed for 1 min. After standing at − 20 °C for 2 h, the mixture was centrifuged at 4000 RCF at 4 °C for 20 min. Then, 300 μl of the supernatant was collected and concentrated by a cryovacuum concentrator. A mixed solution (150 μl, methanol:H_2_O = 1:1, V:V) was added for redissolution, and the supernatant was centrifuged at 4000 r/min for 30 min at 4 °C. The supernatant was removed, and the sample was placed in a sample bottle. Each sample supernatant (10 μl) was mixed with the QC sample to monitor the stability of the LC‒MS analysis process.

A Waters 2D UPLC (Waters, USA) and Q Exactive PLUS high-resolution mass spectrometer (Thermo Fisher Scientific, USA) were used for the separation and detection of metabolites.

### Data preprocessing and analysis of differentially abundant metabolites and pathways

The original mass spectrometry data (RAW files) detected by LC‒MS/MS were imported to Compound Discoverer 3.0 (Thermo Fisher Scientific, USA) for data processing, which mainly included peak extraction, intragroup retention time correction, intergroup retention time correction, adjoint ion combination, missing value filling, background peak labelling, and metabolite identification. The molecular weights, retention times, peak areas, and identification results of the compounds were then derived. Metabolites were identified using the BGI Library, mzCloud, and ChemSpider (HMDB, KEGG, and LipidMaps) databases. The results exported from Compound Discoverer 3.0 were imported into metaX for data preprocessing via the following steps: (1) the probabilistic normalization method (PQN) was used to normalize the data to obtain the relative peak area; (2) quality control-based robust LOESS signal correction (QC-RLSC) was used to correct batch effects; and (3) compounds with a coefficient of variation (CV) greater than 30% of the relative peak area in all QC samples were excluded.

Similarly, R and other software were used for statistical analysis of the metabolite feature table. The screening of differentially abundant metabolites and the search for enrichment pathways were performed using the Metabanalyst linear analysis tool (v5.0, http://www.metaboanalyst.ca).

### Accession number

The original sequence data from the present study have been uploaded and deposited in the NCBI Sequence Read Archive under project number PRJNA859201 and SRA submission number SUB11801292.

## Results

### Influence of *H. pylori* on the microbiota composition of chronic gastritis and gastric carcinoma patients

**Fig. 1:**
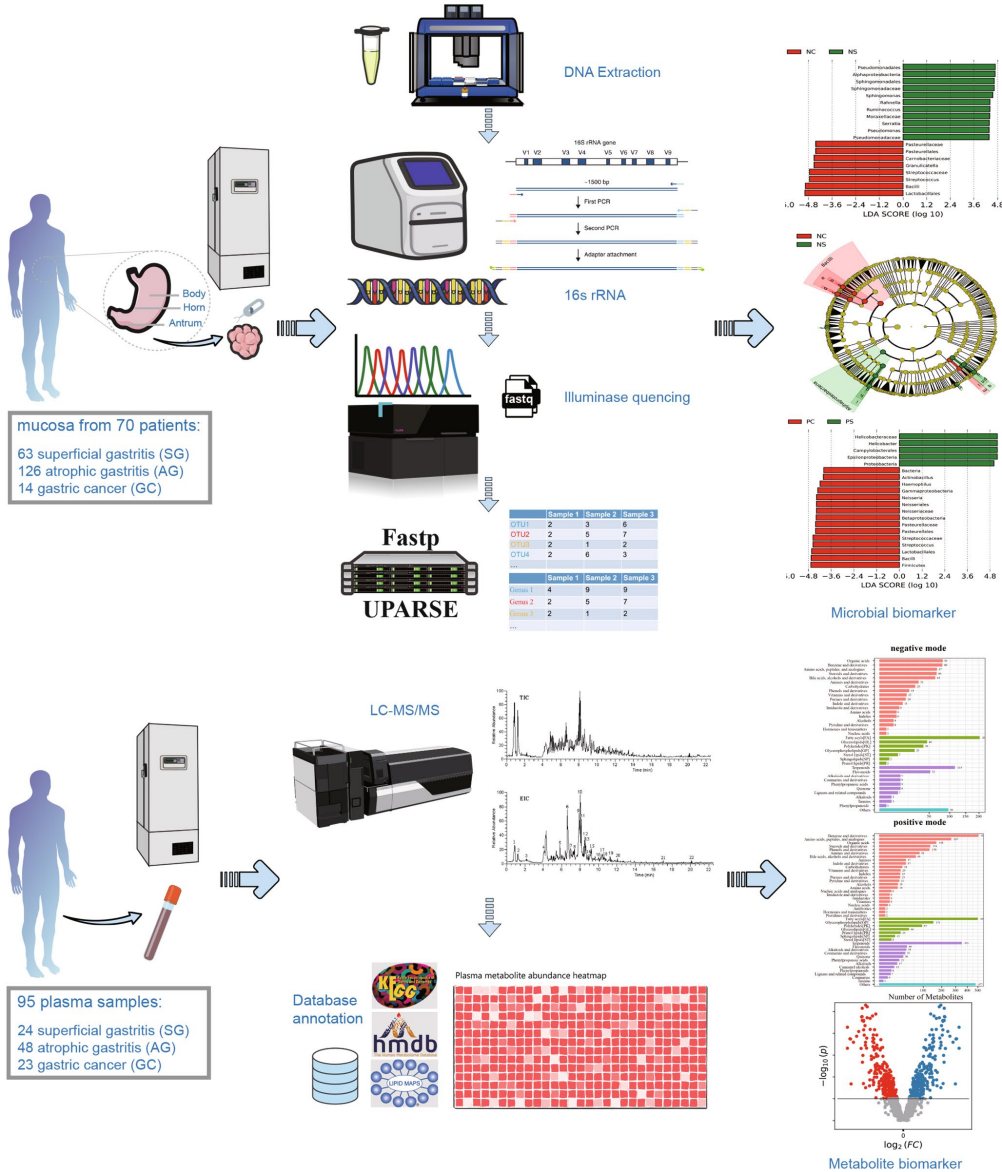
Summary of the study design and experimental flowchart. Sample collection and processing strategy for the microbiome and metabolome analyses.

**Fig. 2:**
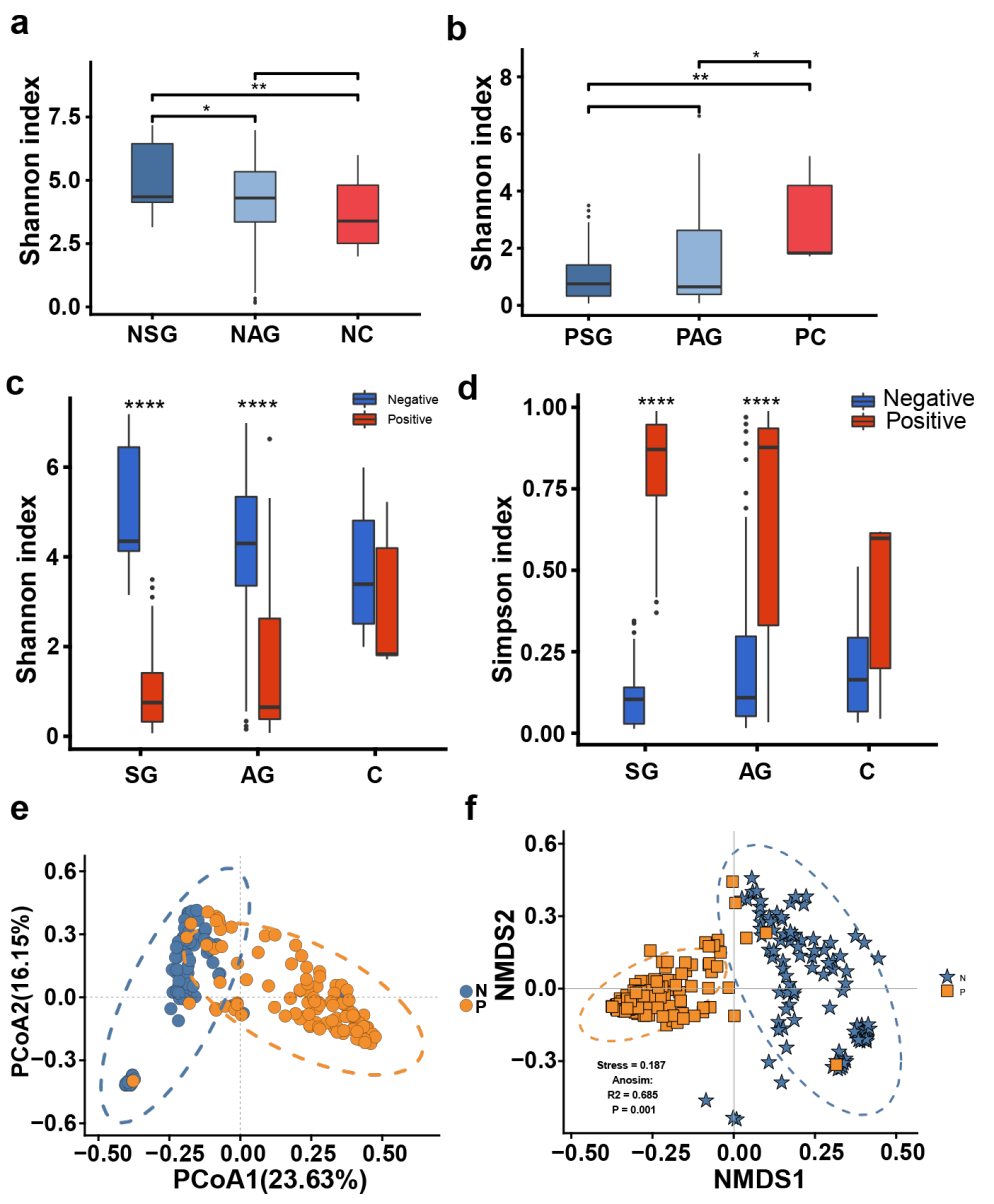
Shannon indices of the gastric microbiota under different pathological conditions in the *H. pylori*^−^ group (**a**) and *H. pylori*^+^ group (**b**). Comparison of the Shannon (**c**) and Simpson (**d**) diversity indices of *H. pylori* infection in different stomach microhabitats. *SG* superficial gastritis, *AG* atrophic gastritis, *C* carcinoma. **e** PCoA clustering of *H. pylori-*positive and *H. pylori*-negative samples. **f** NMDS analysis of *H. pylori-*positive and *H. pylori*-negative samples. ANOSIM analysis of parameter results. N: *H. pylori*-negative, P: *H. pylori*-positive.

A total of 203 gastric mucosa samples from 70 volunteers were ultimately included in the present study after screening. The baseline clinical characteristics of the mucosa samples from the gastric horn, antrum, and body are shown in Supplementary Table 1. The experimental flow chart is shown in Fig. 1. A few confounders of microbiota analyses, such as patient sex (*p *= 0.235), in various stomach microhabitats were not significantly different (Supplementary Table 1). However, there was a significant difference in age (*p* = 0.0141) among patients with different clinical symptoms, and the average age of patients in the gastric carcinoma group was significantly older, which was consistent with previous research. The collected samples were subjected to next-generation high-throughput sequencing. A total of 12,421,783 high-quality reads were obtained via amplicon sequencing, and an average of 54,722 reads per mucosa sample were used for subsequent taxonomic annotation. The species accumulation curves of the OTUs (Supplementary Fig. 1a) in all the samples and the rarefaction curves of the observed species (Supplementary Fig. 1b) in the different groups confirmed that the number of samples collected in the present study was sufficient, indicating that the obtained taxonomic annotation information represented the majority of the identifiable bacteria present in the gastric mucosa. The Venn diagram (Supplementary Fig. 2) indicated common and unique OTUs, with 230 OTUs in the PSG group, 847 OTUs in the PAG group, 11 OTUs in the PP group, and 16 OTUs in the PC group. The PSG and PC groups shared 255 taxa. Moreover, the Venn diagram based on OTUs also showed that there were 773 OTUs in the NSG group, 805 OTUs in the NAG group, 28 OTUs in the NP group, and 4 OTUs in the NC group. The NSG and NC groups shared 269 OTUs. To reveal these differences, multiple α diversity indices were calculated for each sample, and statistical analyses were performed for different groups (Fig. 2, Supplementary Table 2). The Shannon index significantly increased in patients with GC and *H. pylori* infection (*Hp*^+^ group), while in the *Hp*^−^ group, low species richness and low abundance of OTUs were observed in the tumoral microhabitats. Species evenness, such as the Simpson index, decreased in the *Hp*^+^ group with tumours, while in the *Hp*^−^ group, the Simpson index increased. To further investigate whether *H. pylori* colonization alters the community structure of the gastric microbiota in different pathological states, the α diversity of the gastric microbiota was compared in patients with histopathological *Hp*^+^ and *Hp*^−^ strains. Mucosa-associated microbiota in different *H. pylori* statuses showed differences in composition and structure (Fig. 2c, d). Notably, the present data demonstrated that *H. pylori* abundance was negatively correlated with the Shannon index, especially in the gastritis microhabitats (Supplementary Fig. 3), which confirmed the crucial role of *H. pylori* in the community diversity of the intragastric microhabitats.

To explore whether the colonization of *H. pylori* differed among the various groups, the β diversity was calculated using the weighted (quantitative) Bray‒Curtis distance computing method (Fig. 2e). PCoA1 and PCoA2 divided all samples into two clusters between the *Hp*^+^ and *Hp*^−^ groups under pathological conditions of superficial gastritis, atrophic gastritis, and tumoral microbiota. Moreover, nonmetric multidimensional scaling (NMDS) also revealed that *H. pylori* colonization distinguished between the *Hp*^+^ and *Hp*^−^ groups (Fig. 2f, stress = 0.187). A subsequent ANOSIM analysis revealed that there was a significant between-group difference (Fig. 2f, R2 = 0.685, *p *= 0.001).

Taken together, the α- and β-diversity analyses indicated that the community structure in patients with gastritis was altered compared to patients with gastric cancer, and multiple diversity indices were significantly different between patients with and without *H. pylori* infection. The present data demonstrated that in all *H. pylori*-positive samples, the relative abundance of *H. pylori* showed a significant negative correlation with the Shannon index, which confirmed that *H. pylori* infection may be one of the main factors affecting the structure of the bacterial community in the stomach.

### Microbial dysbiosis is associated with gastric carcinoma

**Fig. 3:**
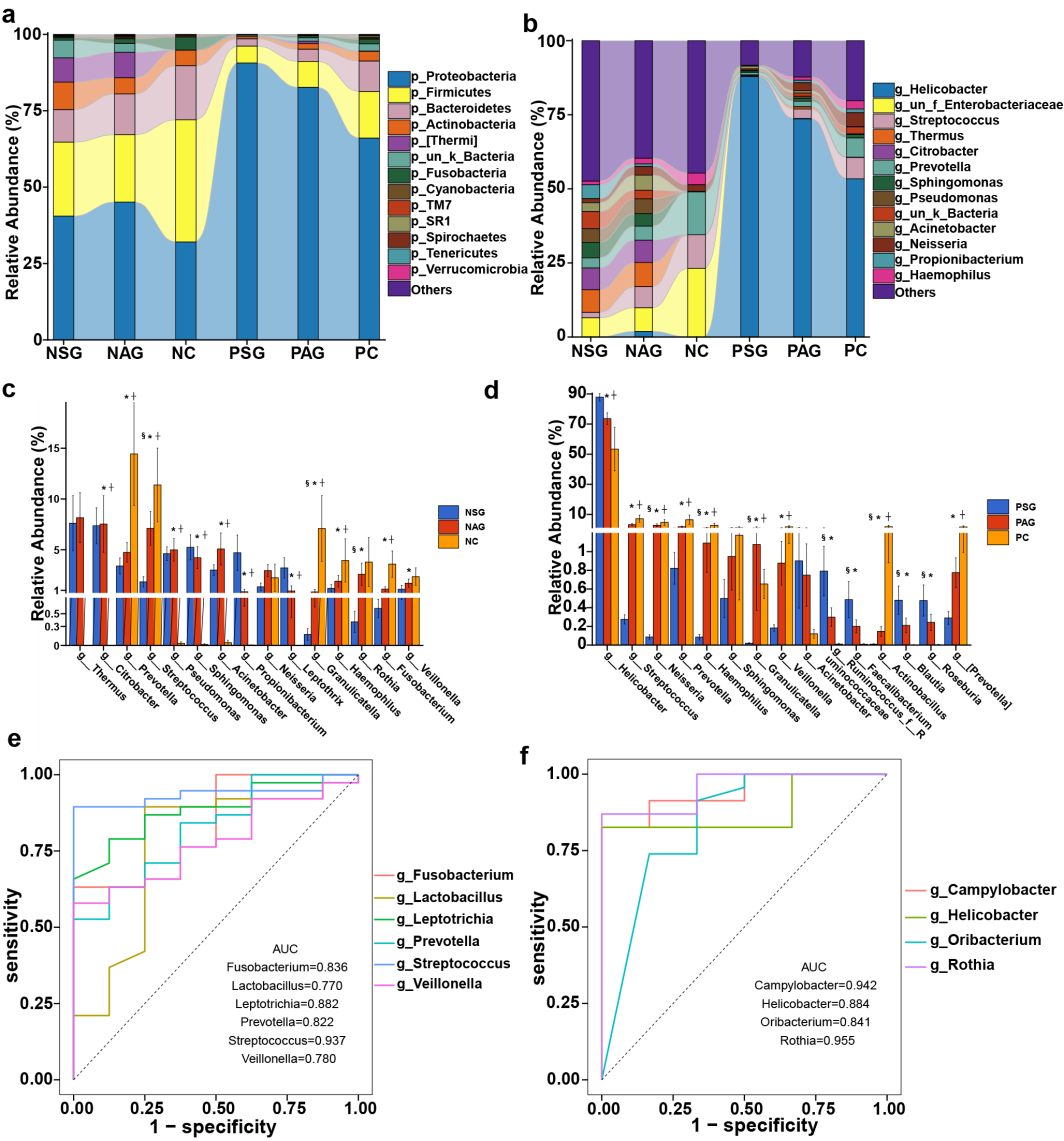
Taxonomic composition of different stomach microhabitats and *H. pylori* infection status at the (**a**) phylum and (**b**) genus levels. Differences in the relative abundances of the 15 most abundant genera at different pathological stages in the (**c**) Hp^−^ group and (**d**) Hp^+^ group. ROC analysis verified the feasibility of these bacterial genera as biomarkers in the Hp^−^ group (**e**) and Hp^+^ group (**f**). SG: superficial gastritis, AG: atrophic gastritis, C: carcinoma. N: *H. pylori* negative, P: *H. pylori* positive. *p < 0.05 between SG and C; ^§^*p *< 0.05 between SG and AG; and ^┼^*p *< 0.05 between AG and C.

To further investigate the taxonomic differences of bacteria in the stomach based on the stage of gastric cancer development, the bacterial annotation information was identified for the stomach microhabitats of superficial gastritis (SG), atrophic gastritis (AG), and gastric carcinoma (GC) patients. At the phylum level, the relative abundances of Firmicutes, Bacteroidetes, Proteobacteria, Actinobacteria, and Fusobacteria were dominant (Fig. 3a, Supplementary Table 3). Although the relative abundances of these phyla were dominant in the three groups, the abundance of Proteobacteria gradually decreased, while that of Firmicutes and Bacteroidetes gradually increased during the development of gastritis and gastric cancer.

At the genus level, *Helicobacter, Streptococcus, Prevotella*, and *Neisseria* abundances were significantly different across all groups. Notably, the relative abundances of *Streptococcus, Prevotella,* and *Granulicatella* significantly increased from the SG and AG groups to the GC group in the *Hp*^−^gastric mucosa microhabitat, while the relative abundances of *Thermus, Citrobacter,* and *Sphingomonas* decreased (Fig. 3b). Compared to the SG and AG microbiota, the relative abundances of*Prevotella* and *Haemophilus* were significantly increased in the GC microbiota, while the relative abundances of *Faecalibacterium* and *Roseburia* were decreased in the *Hp*^+^ gastric mucosa microhabitat in the GC group compared to the SG group (Fig. 3c, d). The genus *Prevotella* significantly colonized the GC microhabitat without *H. pylori*, while *Oribacterium* was significantly more abundant in the GC microhabitat with *H. pylori* (Fig. 3c, d). In contrast to previous reports, the present bacterial annotation data indicated that *Lactobacillus* was enriched in the GC microhabitat without *H. pylori* (Supplementary Fig. 4a). Moreover, the present data confirmed that the relative abundance of *Helicobacter* was substantially decreased during cancer development (Supplementary Fig. 4b). Overall, these results showed that the microbial community structure in the stomach is significantly different between patients with gastritis and patients with GC with or without *H. pylori* infection, as reflected by significant changes in the relative abundances of dominant taxa and specific low-abundance taxa.

Next, 2,330 OTUs were selected to calculate the mean value in the SG group, and the microbial dysbiosis index (MDI) was calculated across all samples. For the *Hp*^−^ samples, the MDI was higher in the GC group compared to the SG group. (*p *< 0.05; Supplementary Fig. 5). In contrast, for *Hp*^−^ patients, a significantly higher MDI was observed in the SG group (*p *< 0.05). This result indicated that the decrease in the MDI was caused by a decrease in the relative abundance of *H. pylori*, which is consistent with the reduced bacterial diversity in *Hp*^+^ patients with SG. In conclusion, the MDI showed an opposite trend between groups with or without *H. pylori* infection.

To explore which taxa contribute more to the three state classifications, LEfSe analysis was performed, and differential taxa were screened among the three patient groups. Pairwise analyses were performed to obtain the LDA scores and *P* values of all taxa (Supplementary Tables 4, 5, 6 and 7), and the top 20 taxa were selected for visualization (Supplementary Fig. 6). In *Hp*^−^ patients, *Sphingomonas, Rahnella, Pseudomonas, Roseburia*, and *Akkermansia* were significantly enriched in the SG group, while *Rothia, Actinocatenispora, Porphyromonas*, and *Bacillus* were enriched in the GC group (Supplementary Fig. 6a). In *Hp*^+^ patients, *Helicobacter, Ruminococcus, Blautia, Faecalibacterium*, and *Bacteroides* were significantly enriched in the SG group, while *Streptococcus, Neisseria, Haemophilus*, and *Veillonella* (Supplementary Fig. 6b) were enriched in the GC group, most of which were potentially pathogenic bacteria in humans and/or animals.

To verify whether the obtained taxa could be used as biomarkers for gastric carcinoma diagnosis, receiver operating characteristic (ROC) curve analysis was performed (Fig. 3e, f). The predictive ability of several genera was assessed using the relative abundances of *Fusobacterium, Lactobacillus, Leptotrichia, Prevotella, Streptococcus,* and *Veillonella* in the GC group without *H. pylori,* as well as *Campylobacter, Helicobacter, Oribacterium,* and *Rothia* in the GC group with *H. pylori*. The abundance of these bacterial genera showed good sensitivity with a high area under the curve (AUC) value (AUC > 0.6).

The present study also explored the discrepancies in gastric microbial communities among the horn, antrum, and body under different disease conditions (SG vs. GC) and *H. pylori* infections. Generally, the bacterial diversity among the three groups was significantly greater in the *Hp*^−^ group at the same anatomical location (Supplementary Fig. 7d). Despite the anatomical location, there was no significant difference in the stomach microbiota among the horn, antrum, and body at the same *H. pylori* status (Supplementary Fig. 7). PCA showed that the samples of the three groups could not be clustered well in the GC group with or without *H. pylori* (Supplementary Fig. 7b). Taken together, wegastric bacterial communities are similar among the three anatomical locations but that the diversity of the microbiota in cancer tissues is significantly different from that in gastric body, horn, and antrum mucosa samples.

### Interactions between community members

**Fig. 4:**
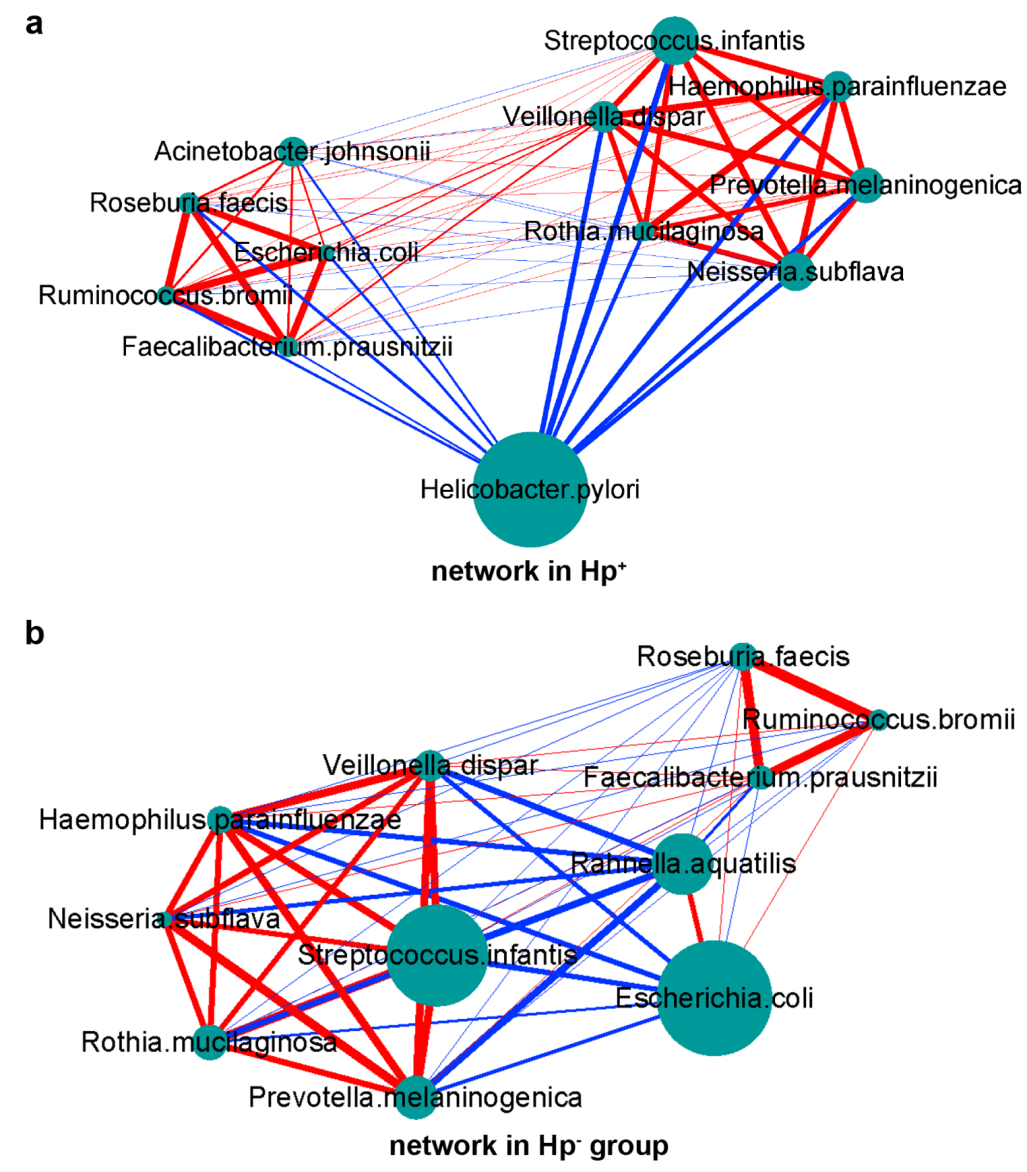
Spearman’s correlation network in the Hp^−^ group (**a**) and Hp^+^ group (**b**). The node area represents the relative abundance of the species. The thickness of the edge indicates the correlation coefficient. Red indicates a positive correlation, and blue indicates a negative correlation.

The differences in the relative abundances of microbiota in the stomach are the result of the interactions and dynamic balance of species in the community. To assess the correlations between species in stomach flora, Spearman’s correlation analysis with Benjamini-Hochberg(BH) adjustments was conducted to calculate interaction matrices based on several top relative abundances of species in all samples (Supplementary Tables 8 and 9), and the results were visualized using Cytoscape software (Fig. 4). Because a previous study [15, 17, 19] has indicated that the colonization of *H. pylori* has a significant influence on the gastric bacterial community, the patients were divided into two groups, namely, the *H. pylori*-infected group and the uninfected group, for the co-occurrence network analysis in the present study.

In the *Hp*^+^ group (Fig. 4a), there was a significant inverse correlation between the relative abundance of *Helicobacter* and that of *Streptococcus, Prevotella, Neisseria,* and *Haemophilus*. Moreover, the relative abundance of *Helicobacter,* such as *Faecalibacterium, Ruminococcus,* and *Roseburia,* was negatively correlated with probiotic populations. These genera, such as *Faecalibacterium, Acinetobacter*, and *Roseburia*, formed a bacterial cluster with strong positive correlations among their internal members.

In the *Hp*^−^ group (Fig. 4b), *Escherichia*, which had a high relative abundance, was inversely correlated with *Streptococcus, Prevotella*, and *Veillonella*. Additionally, *Streptococcus* and *Rahnella* abundances showed a strong negative correlation. These genera showed mainly positive correlations within a bacterial cluster. Similar to those in the *Hp*^+^ group, the abundances of the genera *Faecalibacterium, Ruminococcus,* and *Roseburia* in the gastritis microbiota of the *Hp*^−^ group were strongly positively correlated.

### Inferred functional changes and the predicted bacterial metabolic contribution

Based on the matrix of bacterial abundances obtained from 16S rRNA amplicon sequencing, the functional abundances (COG and KO) of the gastric bacterial communities from each sample were predicted using PICRUSt2 based on the OTU table. Two databases, namely, COG and KEGG, were used to predict the gene functions of the gastric flora (Supplementary Tables 10 and 11), and the results were visualized by STAMP. All differential analyses were corrected by BH adjustment.

In the present study, *H. pylori* colonization was significantly different between the Hp^+^ and Hp^−^groups of the SG, AG, and GC patients for the following COG categories: ribosomal structure and biogenesis; cell wall/membrane/envelope biogenesis; replication; recombination; and repair, (Supplementary Fig. 8). These pathways are generally associated with bacterial cell proliferation and DNA damage repair, suggesting that *H. pylori* may have a high survival ability in the gastric environment and that *H. pylori* colonization may have a wide impact on the proliferation and DNA repair of the gastric microbiota. In particular, several predicted pathways are relevant to bacterial carbohydrate transport and metabolism, and these metabolic reactions have a critical function in the production of short-chain fatty acids (SCFAs). These pathways were enriched in the *Hp*^−^ group, suggesting that *H. pylori* colonization may inhibit short-chain fatty acid-related functional pathways.

**Fig. 5:**
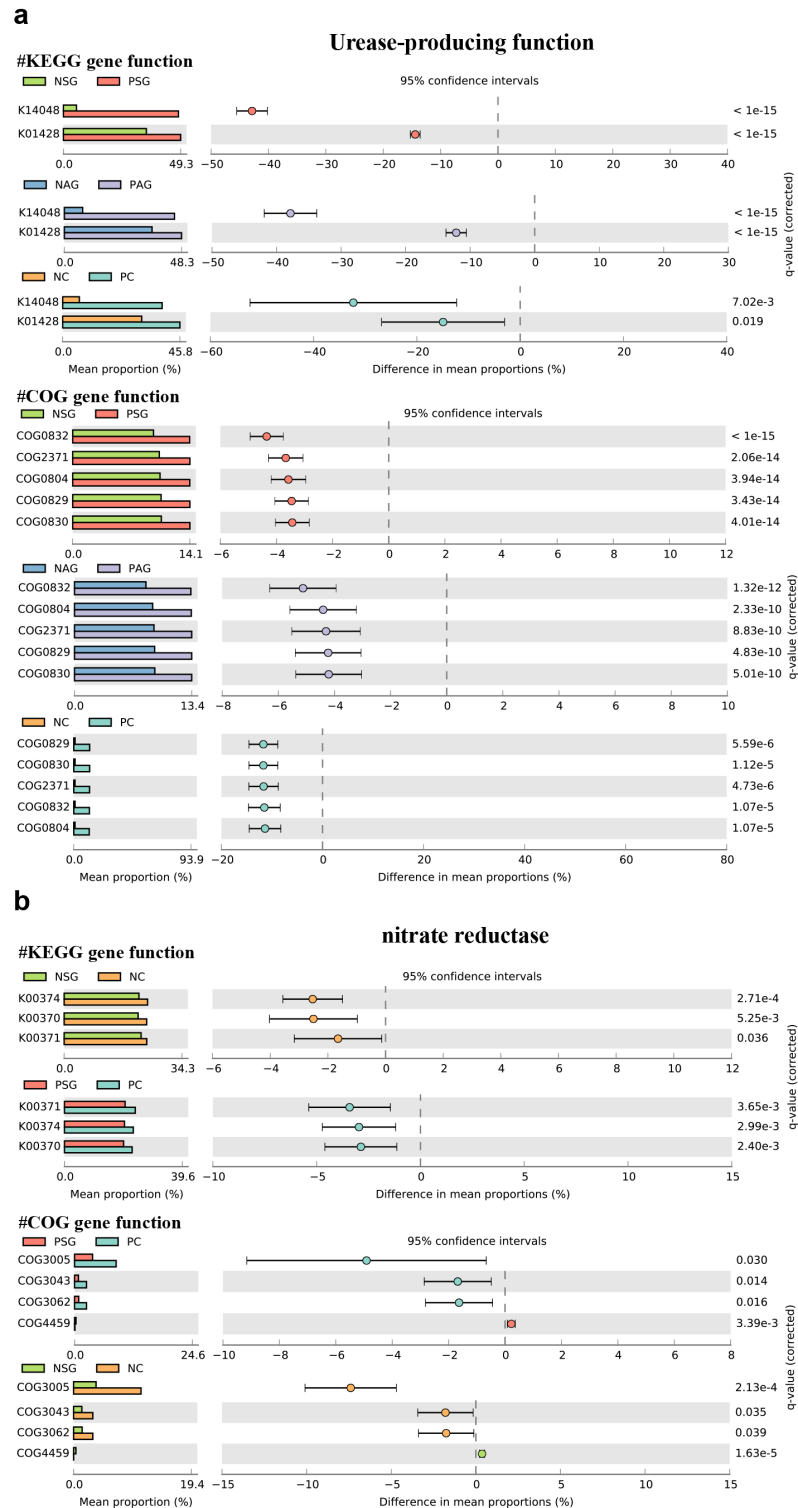
The gastric carcinoma microbiota was characterized by urease and nitrosating bacteria. Functional classification of the predicted metagenome content of the microbiota in chronic gastritis and gastric carcinoma using COG and KO. **a** Differences in predicted urease-producing functions in different groups. **b** Differences in predicted nitrate reductase-producing functions in different groups.

Two functional genes, namely, urease and nitrate reductase, were selected to explore differences in the abundance of genes related to different clinical symptoms and *H. pylori* status (Fig. 5). *H. pylori* neutralizes hydrogen ions around its own cells through ammonia produced by urea decomposition of urease, reducing the acidity of the overall colonization environment, allowing *H. pylori* to survive and colonize the stomach cavity. Consistent with the results of the species composition analysis, multiple KOs associated with urease expression were predicted to increase significantly in the *Hp*^*+*^ group (Fig. 5a). In addition, nitrate reductase-associated KOs were significantly enriched in the GC group (Fig. 5b).

### Analysis of differential metabolites and pathways in the SG and GC groups

A total of 95 plasma samples and 18 QC samples were analysed by LC‒MS/MS via positive (ESI^+^) and negative ion (ESI^−^) modes to improve the coverage of metabolites. The data quality was evaluated through the repeatability of QC sample testing. The base peak chromatograms (BPCs) of the QC samples overlapped well, indicating that the instrument was in a normal state and that the signal was stable throughout the entire process of sample detection and analysis. The percentages of RSD ≤ 30% ion number in the QC samples to total ion number were 94.84 and 94.41% in the ESI^+^ and ESI^−^ modes, respectively. The spectra and ratios demonstrated that the data were of good quality.

After preprocessing, 9,371 and 3,480 peaks were detected in the ESI^+^ and ESI^−^ modes, respectively. Qualitative identification was then performed using publicly available and self-built databases, such as HMDB, KEGG, the BGI Library, and mzCloud (Supplementary Tables 12 and 13). In total, 3,763 metabolites in the ESI^+^ mode and 1,226 metabolites in the ESI^−^ mode were identified and used for subsequent statistical tests (Supplementary Table 14). To adjust the plasma metabolites of extremely high and low concentrations, which may produce systematic differences in subsequent statistical analysis, a quantile normalization of the metabolite characterization table was conducted. All identified metabolites were classified into three categories as follows: compounds with biological roles (the largest number of metabolites in this classification were organic acids in ESI^−^ and benzene and derivatives in ESI^+^); phytochemical compounds (the largest number of metabolites were terpenoids in both modes); and lipids (the largest number of metabolites were fatty acyls in both modes) (Supplementary Fig. 9). Additionally, all these metabolites were identified in the KEGG pathway and classified into six level 1 pathways (such as metabolism and human diseases) with a few level 2 subclasses (Supplementary Fig. 9). In conclusion, these classifications and identifications revealed the overall profile of the metabolites detected in the metabolome.

**Fig. 6:**
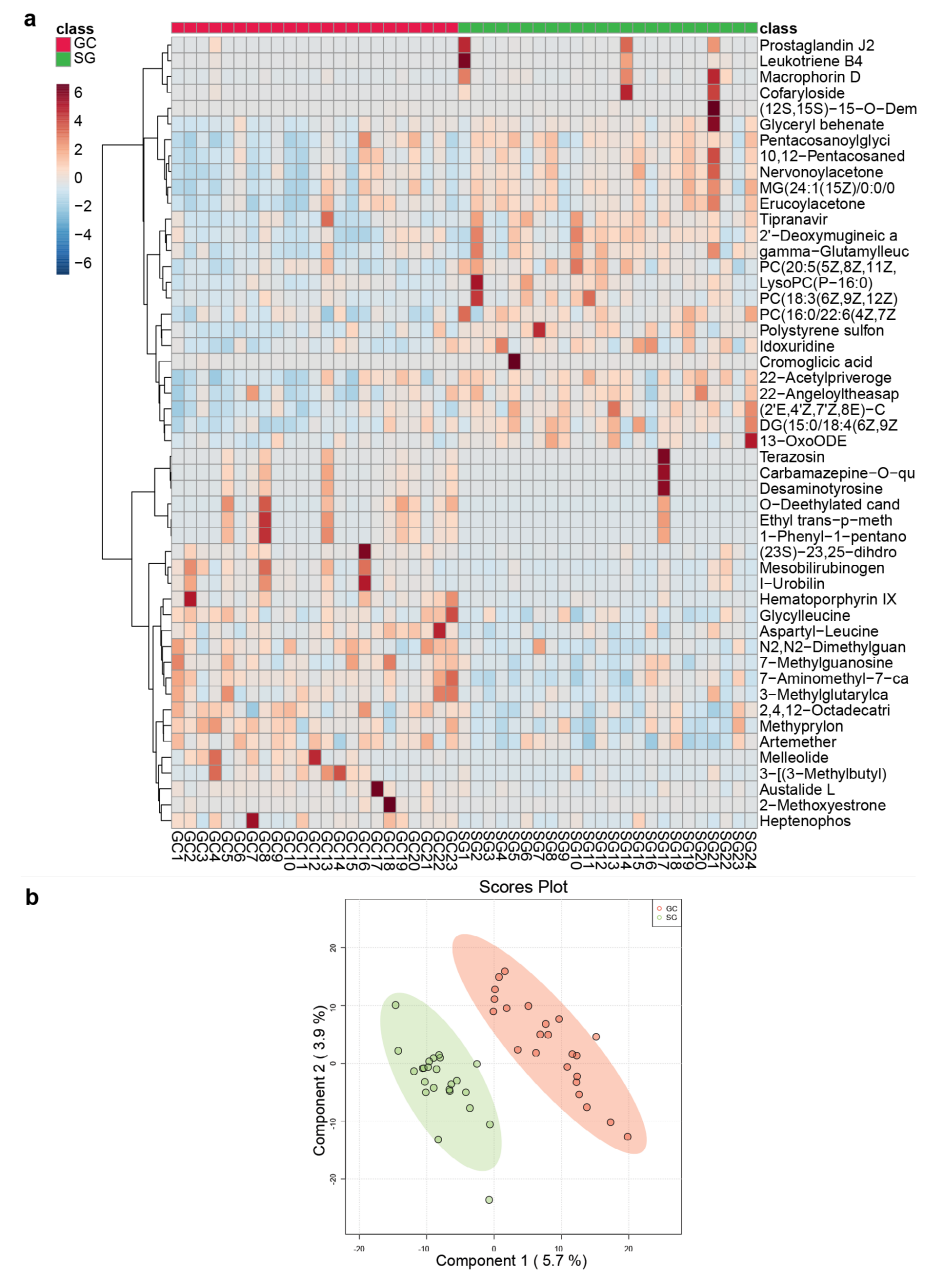
Plasma metabolomics for quantification of metabolites in the gastritis and GC groups. **a** Heatmap of SG and GC samples. Metabolites were clustered and sorted according to *p* value from small to large. **b** sPLS-DA score plot of SG and GC samples.

To explore the differences in the metabolite profiles and search for differentially abundant metabolites with potential effects, heatmap analysis with clustering was performed to evaluate the differences in the metabolite profiles between the two groups, and the Wilcoxon test was used to screen all the metabolites to construct a heatmap (*p *< 0.05). These metabolites were significantly different between the two groups, and had a distinction of colors in the heat map, which differed between the SG and GC groups (Fig. 6a). Moreover, a score plot of the sPLS-DA model was construct, and a heatmap analysis with clustering was performed. The sPLS-DA plot showed a significant separation in all samples between the SG and GC groups, demonstrating the differences in metabolic profiles between the two groups (Fig. 6b). Therefore, these findings indicated that the heatmaps and sPLS-DA models were sufficient to characterize the profile of plasma metabolites and to verify the apparent separation between the SG and GC groups.

**Fig. 7:**
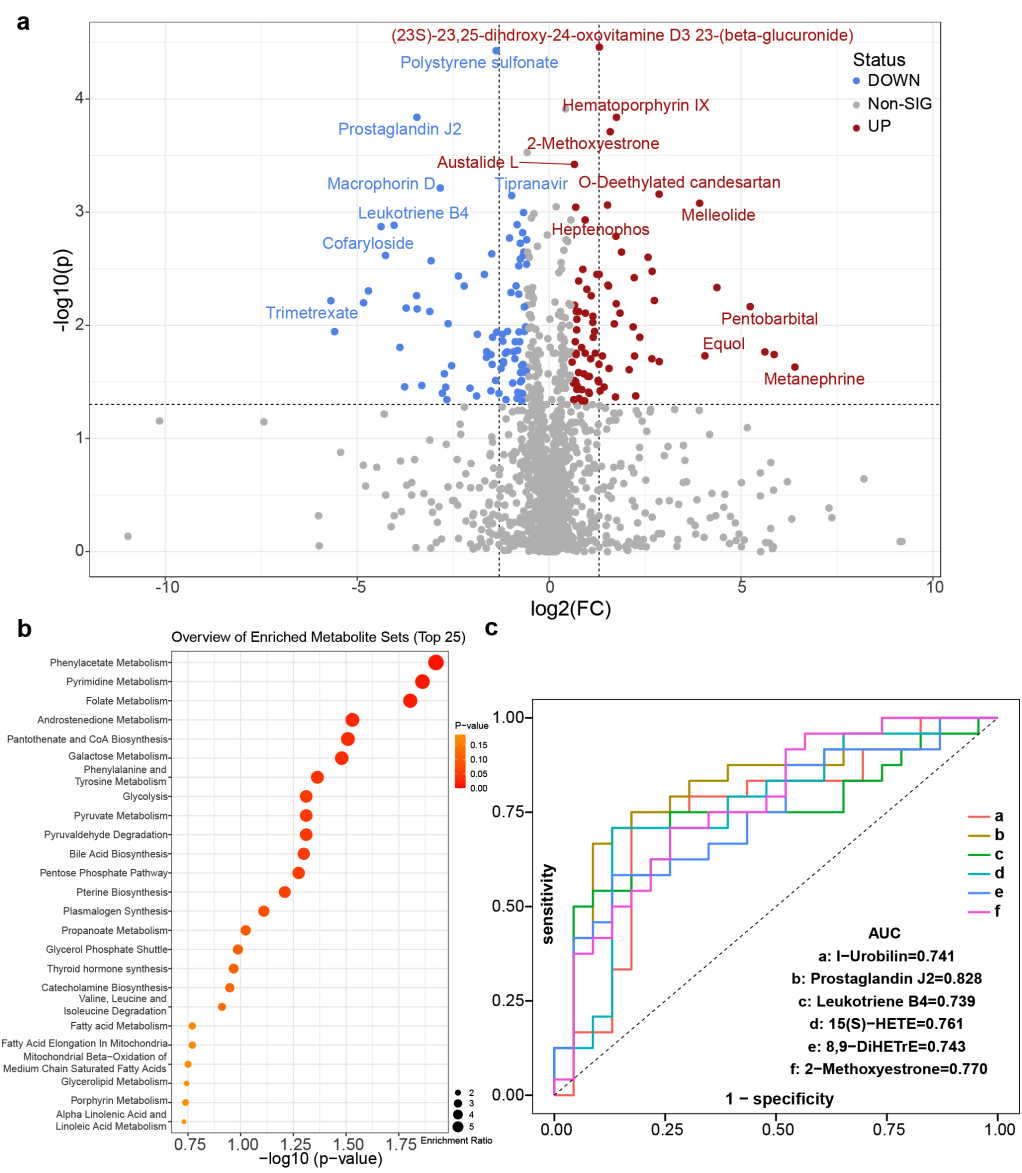
Identification of the differential plasma metabolites and significantly changed metabolites between the SG and GC groups. **a** Volcano plot of the SG and GC groups. Red represented upregulation in the GC group, and blue represented downregulation in the GC group. Grey indicated no significant difference. FC > 1.5, *p *< 0.05. **b** Pathway enrichment analysis of the SG and GC groups. **c** ROC curve analysis verified the feasibility of multiple differentially abundant metabolites as biomarkers between the SG and GC groups. SG: superficial gastritis, GC: gastric carcinoma.

Specific differentially abundant metabolites between the SG and GC groups were further explored. Based on the HMDB, the identified metabolites in the two modes were combined (a total of 1,545), and fold change calculations and Wilcoxon tests were performed. FC values and *P* values were obtained (Supplementary Table 15) and visualized with a volcano plot (FC > 1.5 or < 0.67 and *p *< 0.05 were used as the filtering conditions) (Fig. 7a). In total, 81 differentially abundant metabolites were upregulated in the GC group, including metanephrine, 2-methoxyestradiol, equol, 2-indolecarboxylic acid, and alpha-N-phenylacetyl-L-glutamine, whereas 86 differentially abundant metabolites were downregulated in the GC group, including 8,9-DiHETrE, leukotriene B4, prostaglandin J2, 15(S)-HETE, and 15(S)-hydroxyeicosatrienoic acid. Moreover, metabolic pathway enrichment analysis of differentially abundant metabolites was performed based on the SMPDB, which revealed that a few metabolic pathways (*p *< 0.05) were significantly enriched (Fig. 7b), such as phenylacetate metabolism, pyrimidine metabolism, and folate metabolism.

To verify whether the obtained differentially abundant metabolites could be used as markers to distinguish SG from GC in clinical diagnosis, six differentially abundant metabolites with potential predictive ability were selected for receiver operating characteristic (ROC) curve analysis based on *p* values and FC values. By calculating the integral area of the curve, the differentially abundant metabolites with an AUC value > 0.7 were considered candidate markers (Fig. 7c). The AUC values showed that prostaglandin J2 and 2-methoxyestrone efficiently distinguished GC patients from SG patients.

### Analysis of differential metabolites and pathways in the NAG and PAG groups

Atrophic gastritis is a common condition with multiple pathogenic factors, and it exhibits no significant specificity in clinical patients. However, atrophic gastritis has potential to develop into intestinal metaplasia or precancerous lesions. Globally, *H. pylori* infection is common in the atrophic gastritis stage due to its potential cytotoxicity and genotoxicity. To investigate whether colonization by *H. pylori* had a significant effect on the plasma metabolite profile, the same data preprocessing and statistical analysis were performed in the NAG and PAG groups.

**Fig. 8:**
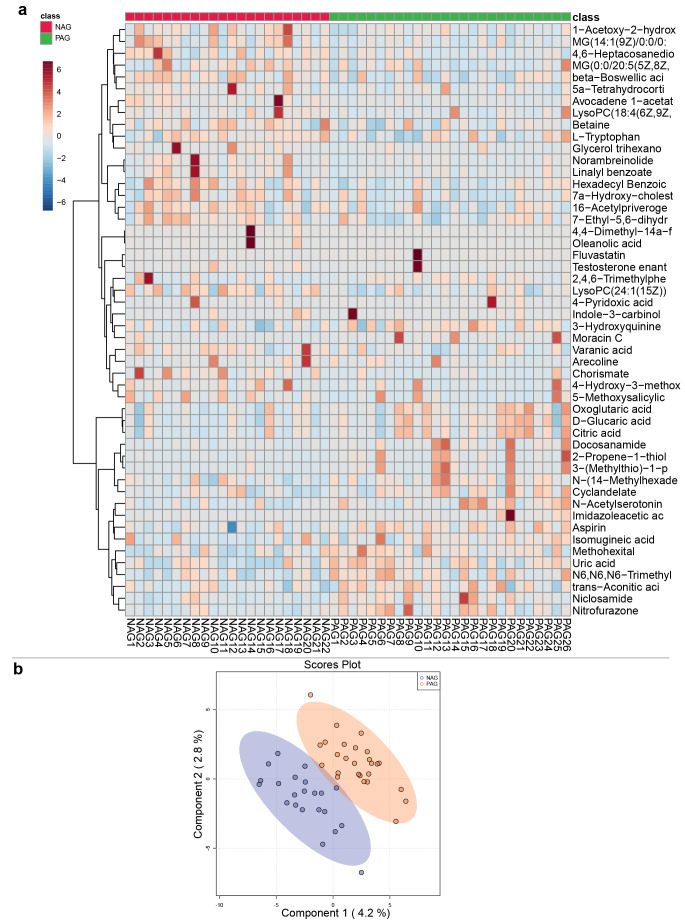
Plasma metabolomics for the quantification of metabolites in the Hp^−^ and Hp^+^ groups. **a** Heatmap of NAG and PAG samples. Metabolites were clustered and sorted according to *p* value from small to large. **b** sPLS-DA score plot of NAG and PAG samples.

Heatmap analysis with clustering was performed to evaluate the differences in metabolite profiles between the two groups, and the Wilcoxon test was used to screen all the metabolites to construct a heatmap (*p *< 0.05), which demonstrated that these metabolites were significantly different between the two groups (Fig. 8a). A score plot of the sPLS-DA model was conducted, a heatmap analysis with clustering was performed. The sPLS-DA plot showed a significant separation in all samples between the NAG and PAG groups, demonstrating the differences in metabolic profiles between the two groups. (Fig. 8b). The metabolites were clustered into three clusters, and two of them had different “heat” colours between the NAG and PAG groups. Therefore, these results indicated that heatmaps and the sPLS-DA model were also sufficient for characterizing the profiles of plasma metabolites and confirming the apparent differences between the NAG and PAG groups.

**Fig. 9:**
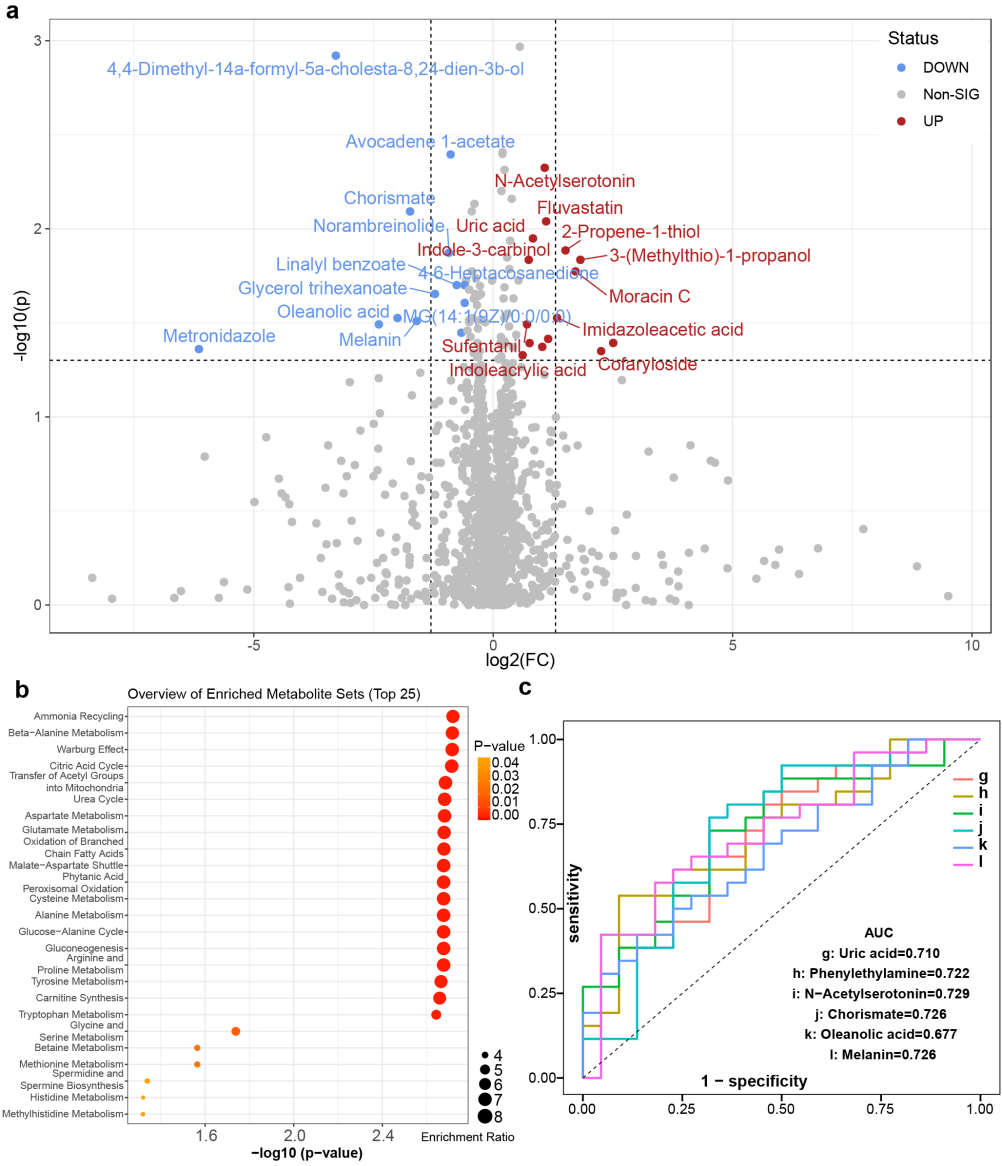
Identification of the differential plasma metabolites and significantly changed metabolites between the NAG and PAG groups. **a** Volcano plot of the NAG and PAG groups. Red represents upregulation in the PAG group, and blue represents downregulation in the PAG group. Grey indicates no significant difference; FC > 1.5, *p *< 0.05. **b** Pathway enrichment analysis of the NAG and PAG groups. **c** ROC curve analysis verified the feasibility of multiple differentially abundant metabolites as biomarkers between the NAG and PAG groups. NAG: atrophic gastritis with *H. pylori* negative, PAG: atrophic gastritis with *H. pylori* positive.

Based on the HMDB, the identified metabolites in the two modes were combined (a total of 1545), and fold change calculations and Wilcoxon tests were performed. The FC and *P* values were obtained (Supplementary Table 16) and visualized with a volcano plot (FC > 1.5 or < 0.67 and *p *< 0.05 were used as the filtering conditions) (Fig. 9a). Overall, 15 differentially abundant metabolites, such as N-acetylserotonin, uric acid, imidazoleacetic acid, phenylethylamine, and indoleacrylic acid, were upregulated in the PAG group, while 13 differentially abundant metabolites, such as chorismate, oleanolic acid, melanin, and 3,4-dihydroxyphenylglycol, were downregulated in the PAG group. In addition, metabolic pathway enrichment analysis of differentially abundant metabolites was performed based on SMPDB, and a few metabolic pathways (*p *< 0.05) were significantly enriched (Fig. 9b), such as ammonia recycling, beta-alanine metabolism, and folate metabolism.

Furthermore, six differentially abundant metabolites with potential predictive ability were selected for receiver operating characteristic (ROC) curve analysis based on *p* values and FC values. By calculating the integral area of the curve, the differentially abundant metabolites with an AUC value > 0.7 were considered candidate markers (Fig. 9c). The curves indicated that N-acetylserotonin and chorismate efficiently distinguished the PAG and NAG groups.

## Discussion

Previous studies have reported conflicting results on gastric microbiome dysbiosis across the GC cascade. Incomplete coverage of research subjects and a single research design make it difficult to generate statistically significant conclusions [22, 30]. In the present study, *H. pylori* colonization caused microbiota dysbiosis, which was consistent with the overrepresentation of potential pathogenic bacterial genera and metabolites characterized by the induction of inflammatory and immune dysregulation, leading to genetic instability and cancer. In particular, the present multiomics analysis identified GC-derived microbes and metabolic biomarkers associated with gastritis, including the gastric precancerous cascade, which might be useful and suitable for long-term monitoring of the risk of GC.

Recent evidence indicates changes in microbial diversity and function in gastric microbiota profiles during cancer development. To date, however, the interaction between gastric microbiota dysbiosis and gastric tumorigenesis is still unclear [31]. The present findings confirmed that the composition of the gastric microbiota was altered in the presence of *H. pylori* infection, which might increase the risk of developing gastric cancer. Similar to other studies, *Helicobacter* was detected as the most abundant genus in *H. pylori*-positive samples, while in *H. pylori*-negative samples, *Helicobacter* was characterized by an increase in genera that colonize the oral cavity or upper respiratory tract, such as *Streptococcus, Prevotella,* and *Neisseria*. Recently, some studies have indicated that the elimination of *H. pylori* may reduce the incidence of gastric cancer [13, 32]. Furthermore, the present study revealed that *H. pylori* was significantly decreased in the tumoral microbiota, consistent with the finding that GC-specific microbiota were characterized by a decreased abundance of *H. pylori* and enrichment of other microbes, such as *Granulicatella, Ruminococcus,* and *Thermus.* Previous studies had reported that gastric bacteria-mediated carcinogenesis might mechanistically depend on the loss of specialized glandular tissue and decreased acid secretion, allowing the overrepresentation of other commensals in less acidic environments [15, 16, 33]. As a carcinogenic pathogen, *H. pylori* may participate in procarcinogenic events by eliciting an immune response in gastric epithelial cells, especially an imbalance of Treg/Th17 cells [34]. However, whether *H. pylori* can function as a bacterial driver and interact with non-*H. pylori* gastric bacteria remains unknown. Although the potential role of the commensal microbiota in carcinogenicity has not yet been clarified, *H. pylori* colonization in the stomach alone is not sufficient to induce gastric carcinogenesis [14–16]. Lofgren et al. [33]. demonstrated that *H. pylori*-induced GC was promoted by crosstalk and cooperation between *H. pylori* and other commensal flora. The genera *Streptococcus* and/or *Prevotella,* which are also associated with numerous tumours, are potential risk factors for GC [35, 36]. Moreover, the causal relationship between *Streptococcus* spp. and colorectal and gastric cancer has been confirmed in a series of cohort studies. Chen et al. [37] verified a risk association of *Streptococcus* in upper digestive tract tumours by comparing 87 oesophageal squamous cell carcinoma patients with 85 healthy controls. Shen et al. [38, 39] reported that *Streptococcus* is the most dominant genus in the stomach in the absence of *H. pylori*. The abundance of *Actinobacillus*, an oral and respiratory pathogen, is also increased in the tumoral microhabitat [40]. Together with the present results, these findings indicated that one specific bacterium alone was not sufficient for carcinogenesis and that the overgrowth of pathogenic bacteria and dysbiosis-driven disruption of immune and inflammatory responses might be essential for GC pathogenesis.

Strains of *Lactobacillus, Bifidobacterium,* and *Saccharomyces* have long been used as potential probiotics and therapeutic agents, and Roseburia spp.*, Akkermansia *spp*., Propionibacterium *spp., and *Faecalibacterium* spp. have shown potential and promising application in the future [41–43]. Furthermore, these commensal bacteria, especially *Roseburia* spp. and *Akkermansia *spp., are butyrate-producing bacteria, and they exert their fundamental benefits through anti-inflammatory, barrier protective, and immune maintenance mechanisms. Notably, *Roseburia *spp. may also serve as biomarkers for symptomatic pathologies or as probiotics for the restoration of beneficial flora. Previous studies have reported that the proportion of *Roseburia* significantly decreases in patients with IBD and alleviates colitis pathology by reducing the percentage of Th17 cells and maintaining immune balance and the intestinal epithelial barrier [41, 43]. The present study showed that the relative abundances of *Faecalibacterium* and *Roseburia* decreased gradually during superficial gastritis to gastric cancer in the gastric mucosa microhabitat. Sokol et al. [15, 33]. reported that a reduction in the abundance of a highly butyrate-producing bacterium, *Faecalibacterium prausnitzii*, was associated with a greater risk of postoperative cancer recurrence and chronic inflammation. Although the role of *Faecalibacterium* has been characterized in ulcerative colitis (UC), Alzheimer’s-type dementia, and liver cirrhosis, only small cohorts of patients with gastric disease have been studied, and conflicting data have been reported [42, 44]. Consistent with the present conclusion, previous studies have demonstrated an increased abundance of *Lactobacillus* in GC. Considering the wide use of *Lactobacillus* in the food industry as a beneficial organism for centuries, it is surprising that the bacterium is enriched in GC but rarely present in healthy subjects [45]. *Lactobacillus* produces metabolites, such as lactate, which may serve as an energy source for tumour growth and angiogenesis. Moreover, *Lactobacillus* species reduce nitrate to nitrite, which leads to the formation of large amounts of N-nitroso compounds [46]. It is highly important to clarify whether *Lactobacillus* enrichment has protumourigenic effects or acts only as a marker for bacterial overgrowth during disease progression. To better clarify the role of *Lactobacillus* in the development of GC, further studies at the strain and species levels need to be performed.

After analysing the microbial composition, diversity, and features associated with gastric carcinoma, the functional features of the microbiota were investigated. In addition to *H. pylori*, urease-producing (UB) non-*H. pylori* microbes, including *Actinomyces, Clostridium, Corynebacterium, Enterococcus, Streptococcus, Staphylococcu,* and *Yersinia,* predominantly reside in the oral cavity, gastrointestinal tract, and skin [15, 16, 47]. As expected, the present data showed that the abundance of UB was significantly greater in *H. pylori*-positive cancer samples than in *H. pylori*-negative cancer samples. Urease protects bacteria from gastric acidity to facilitate their colonization by modifying bacterial substrate availability and local immune responses. Therefore, bacteria buffer gastric acidity possibly through their ability to produce NH_3_ [16, 48]. Specifically, the present findings indicated that the microbiota had increased nitrate reductase activity in gastric carcinoma patients compared to chronic gastritis patients, supporting the hypothesis that dysbacteriosis in the stomach mucosa resulted in attenuated gastric acidity during carcinogenesis, allowing opportunistic pathogens to reduce nitrate to nitrite, a precursor of carcinogenic N-nitroso compounds. Urease-producing and nitrate-reducing bacteria synergistically contributed to the release of procarcinogenic factors and shaped the tumour microenvironment.

Cancer cells exhibit a network of altered cellular metabolism pathways, which provides a biochemical basis and contributes to tumour growth. Therefore, cancer metabolism has recently become a considerable research focus and provides therapeutic guidance. Many metabolomics studies have reported changes in metabolites in the plasma, urine, and tissue of gastric cancer patients. However, systematic validation of cancer metabolism is lacking [49]. The aim of the present study was to investigate plasma metabolic features and identify candidate biomarkers for gastric cancer. The pathway analysis results of the two considered models were depicted in Fig. 6, revealing a number of specific metabolic pathways. Lipid metabolism becomes dysregulated because cancer cells survive by enhancing exogenous lipid uptake and activating endogenous lipid synthesis to supply energy and meet the need for rapid proliferation [50]. Consistent with this conclusion, the present study demonstrated that lipid metabolites increased significantly. Assessment of the fasting lipid profile indicated that upregulated levels of 8,9-DiHETrE and 15-HETE were detected in the plasma of GC patients. Specifically, the abundances of *Streptococcus* and *Prevotella* were positively associated with abnormal lipid metabolism in GC patients. By comparing the SG and GC groups, the present study showed that phenylacetate and phenylacetylglutamine metabolism were increased in GC patients, which was in agreement with the results of a previous study using untargeted metabolomic analysis [51]. Additionally, the present study indicated that the development of GC was associated with elevated levels of plasma phenylacetylglutamine, which was involved in microbial phenylalanine or glutamine metabolism. Therefore, the interaction of microbiota-derived metabolites with their host may unravel the aetiology of GC [52]. Further analysis revealed that I-urobilin, prostaglandin J2, leukotriene B4, methoxyestrone, and HETE may be prognostic factors for GC. The present study identified four novel metabolites that have been investigated in other diseases, but their pathophysiological role in the development of GC has not been reported. For example, 2-methoxyestradiol is a promising anticancer agent for cancer therapy [53]. The demethylation of 2-methoxyestrogens may be due to the action of microbial enzymes *in vivo*; however, the pathophysiological role of 2-methoxyestrogens has not been reported [54]. Another elevated metabolite, metanephrine, plays major roles in regulating important cellular processes, including cell proliferation, metabolism, differentiation, and protein synthesis [55]. However, the specific functions and future prospects of metanephrine have not been explored. Previous studies have suggested a link between *H. pylori* and metabolic abnormalities [39]. In the present study, comparison of the metabolic profiles of *H. pylori*-infected and uninfected individuals revealed significant differences between the two groups. The present data highlighted disturbances in the metabolism of uric acid, energy, amino acids, and lipids in the plasma of humans with superficial gastritis attributed to *H. pylori* infection to a certain extent. Moreover, a previous study on the relationship between *H. pylori* infection and metabolic abnormalities had revealed that *H. pylori* infection was associated with increased uric acid levels [56], which was consistent with the present study (Fig. 7). Thus, these findings suggested that uric acid accumulation in human plasma was closely related to the pathogenesis of *H. pylori* infections. Importantly, the present study indicated that *H. pylori* infection might perturb the gastric microbiota and homeostasis, subsequently causing systemic metabolic disorders. Because the role of the different bacteria of the gastric microbiota in the process of gastric carcinogenesis remains unclear, future experiments need to validate the accuracy and sensitivity of the diagnostic biomarkers for GC, as well as elucidate the molecular mechanism and key mediators responsible for the specific metabolism in GC.

## Conclusion

The present studies conclude that *H. pylori* colonization influences the overall structure of the gastric microbiota in gastritis and cancer microhabitats. *Streptococcus, Prevotella,* and *Granulicatella* are candidate pathogenic bacterial species in *H. pylori*-negative patients. The gastric microbiota profile of patients with carcinoma is significantly different from that of patients with chronic gastritis. Alterations in the microbial community composition, function, and ecological network in GC tissues may be involved in carcinogenesis and are consistent with increased genotoxic potential. Evaluation of the plasma metabolite profiles indicates that metabolite alterations may be involved in pathological responses. The present analysis of mucosa-associated microbiota and plasma from GC- or *H. pylori*-infected patients may contribute to the determination of potential biomarkers for diagnosis and improve the surveillance of gastric cancer patients via minimally invasive analysis. In addition, these findings may also provide new therapeutic targets for the development of novel treatment approaches in clinical practice.

## Electronic supplementary material

Below is the link to the electronic supplementary material.

Supplementary Material 1

Supplementary Material 2

Supplementary Material 3

Supplementary Material 4

Supplementary Material 5

Supplementary Material 6

Supplementary Material 7

Supplementary Material 8

Supplementary Material 9

Supplementary Material 10

## Data Availability

The original sequence data from this study have been uploaded and deposited in the NCBI Sequence Read Archive under project number PRJNA859201 and SRA submission number SUB11801292. https://submit.ncbi.nlm.nih.gov/subs/bioproject/SUB11801292/overview.
