# Supplementary figures and images for: Comprehensive multiomics analysis of the signatures of gastric mucosal bacteria and plasma metabolites across different stomach microhabitats in the development of gastric cancer
Source: Cell Oncol (Dordr). 2024 Jul 4;48(1):139–59. doi: 10.1007/s13402-024-00965-3 (PMC11850404; doi:10.1007/s13402-024-00965-3)

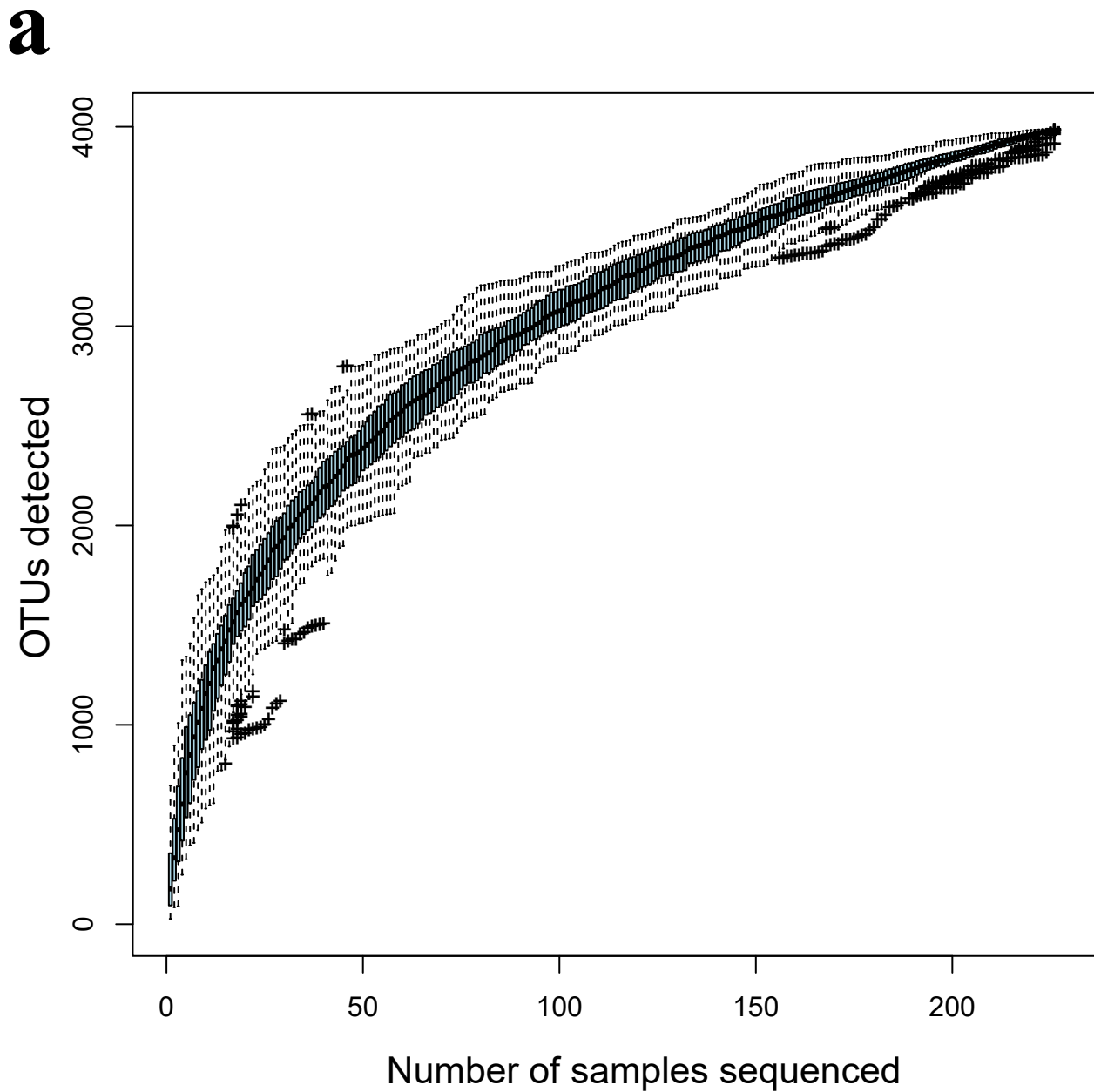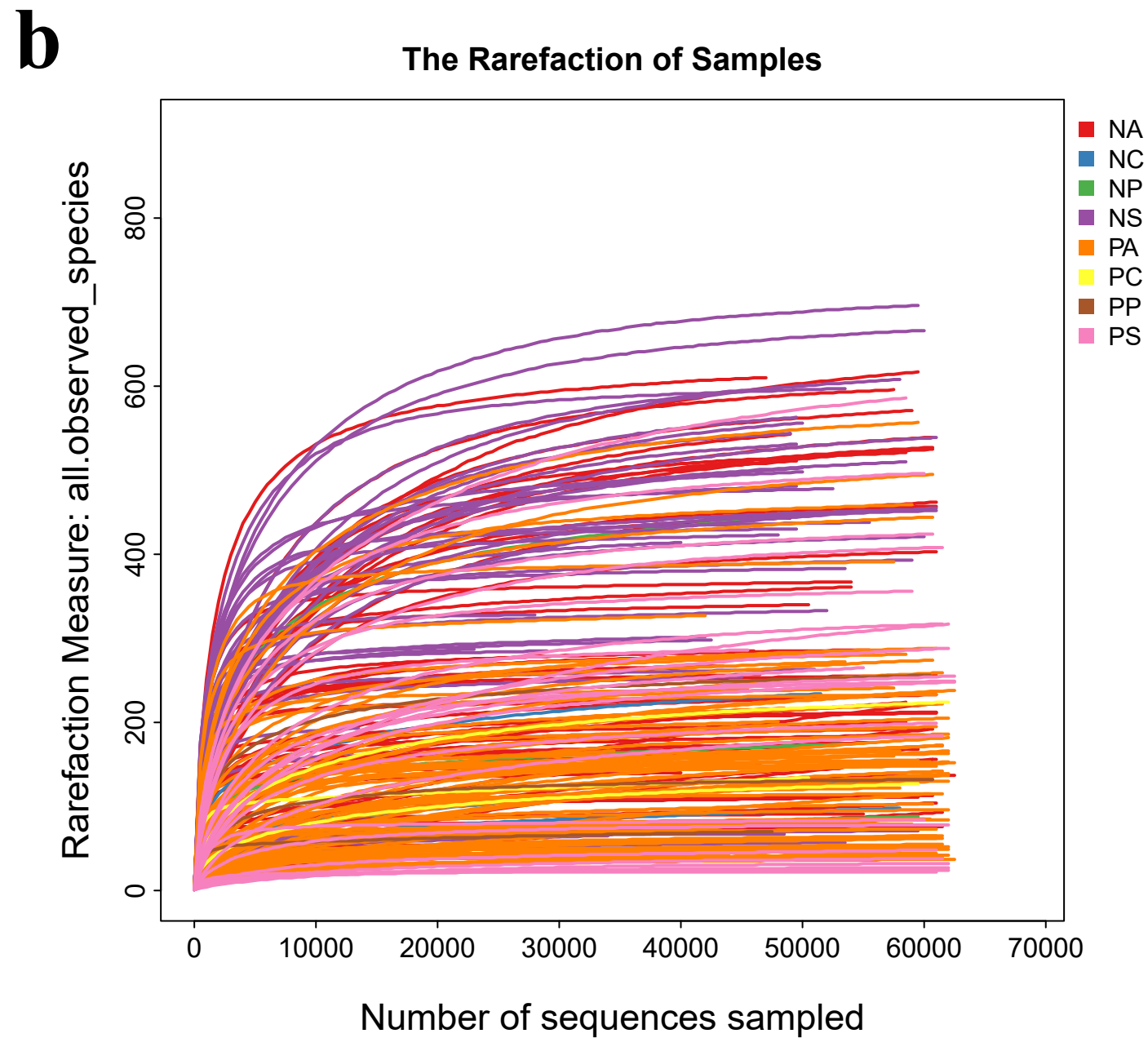

Supplement: Supplementary file 1 — Supplementary Material 1 [file 13402_2024_965_MOESM1_ESM.pdf]

**a**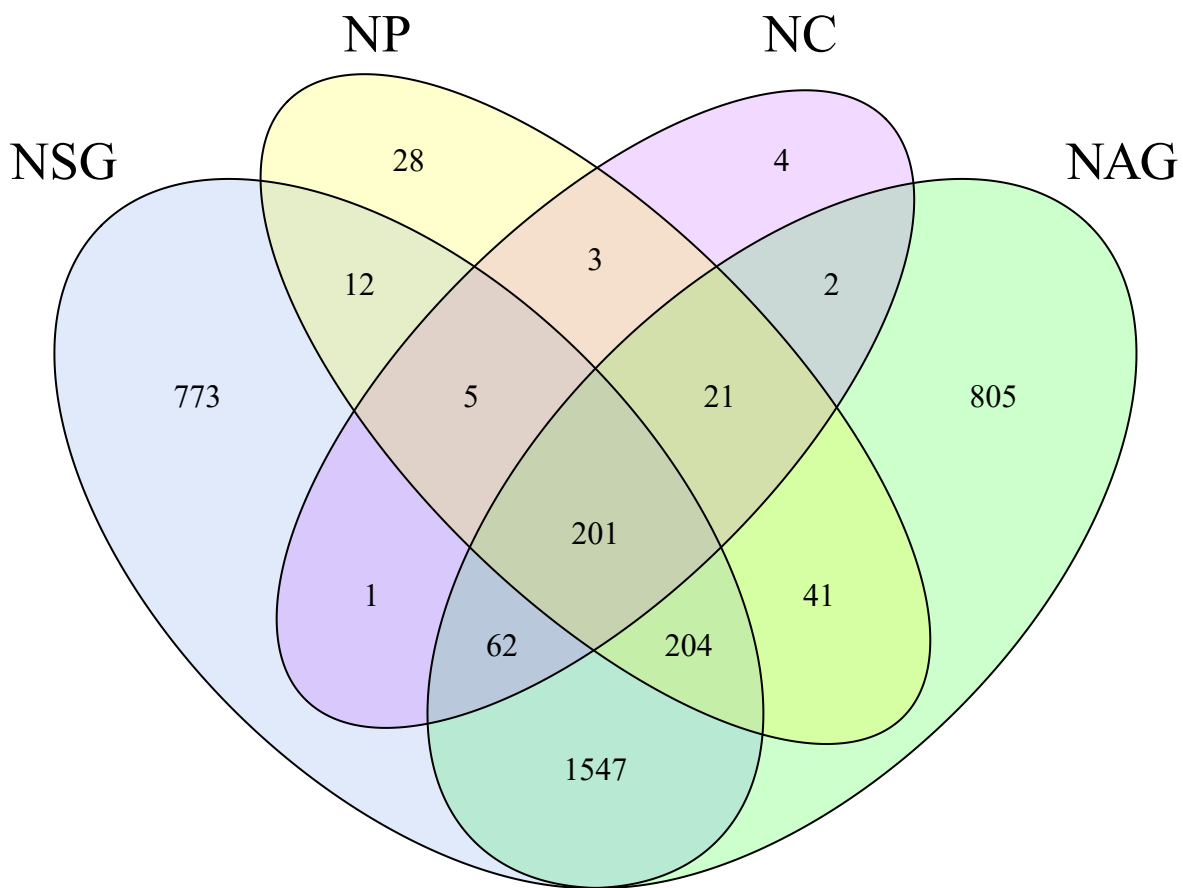**b**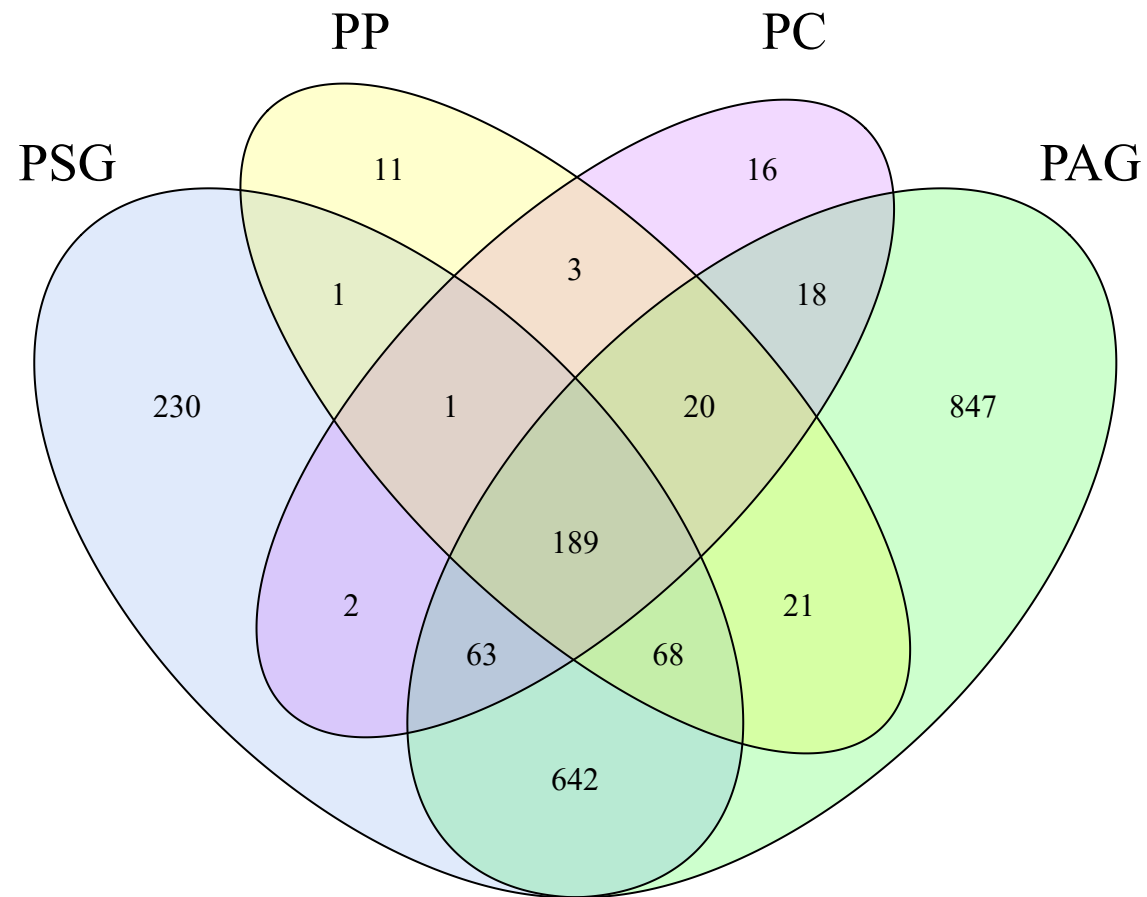

Supplement: Supplementary file 2 — Supplementary Material 2 [file 13402_2024_965_MOESM2_ESM.pdf]

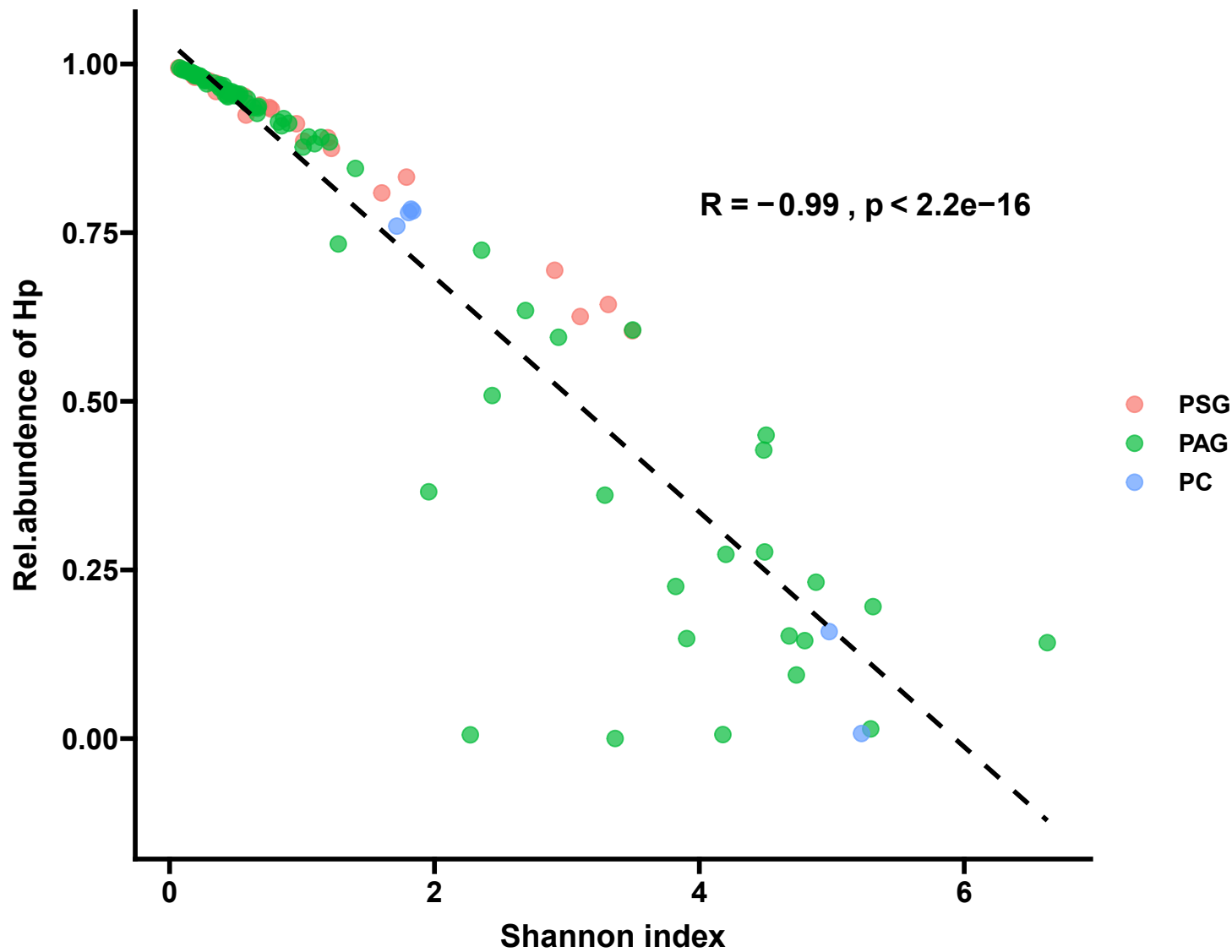

Supplement: Supplementary file 3 — Supplementary Material 3 [file 13402_2024_965_MOESM3_ESM.pdf]

**a**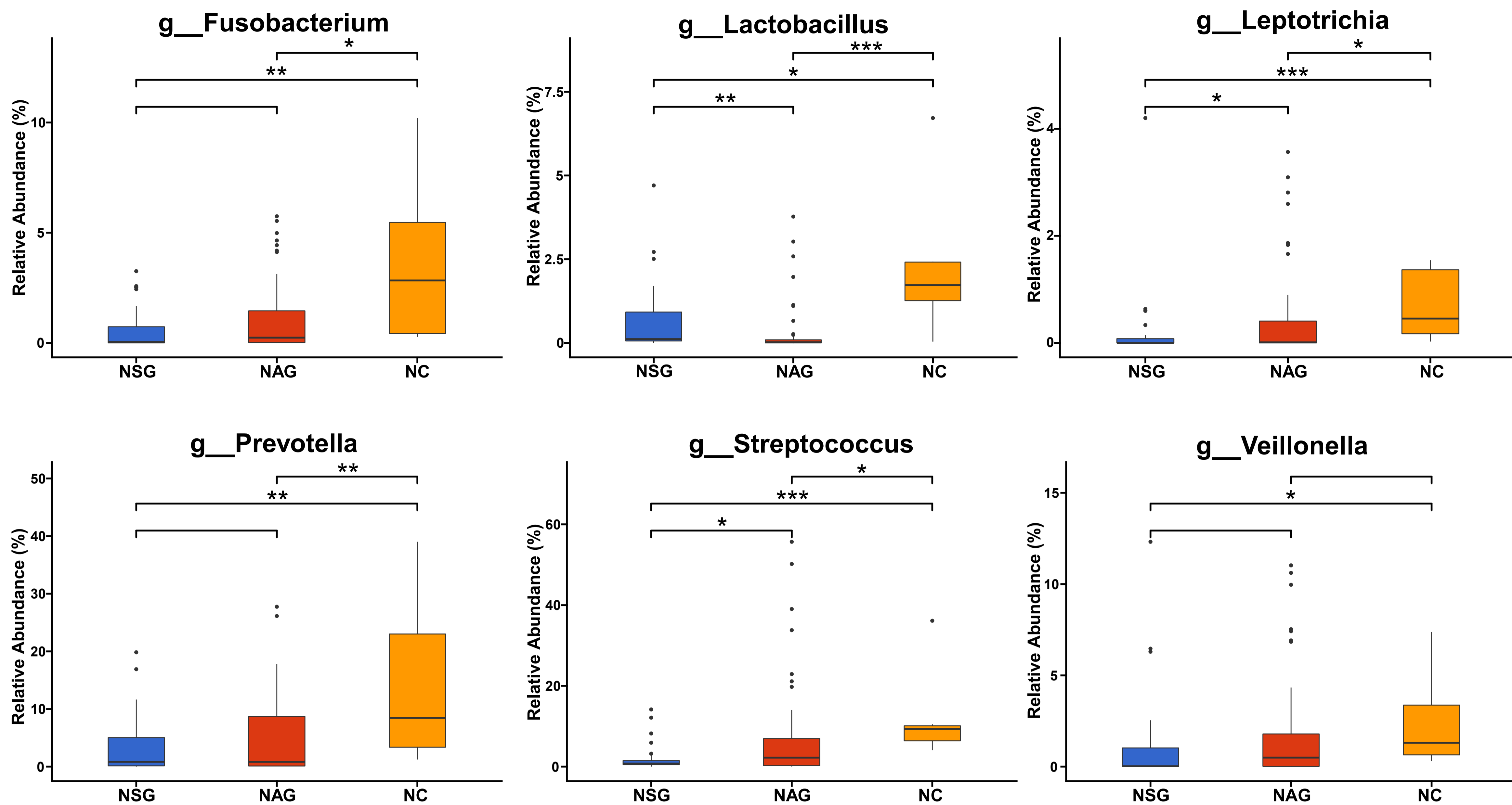**b**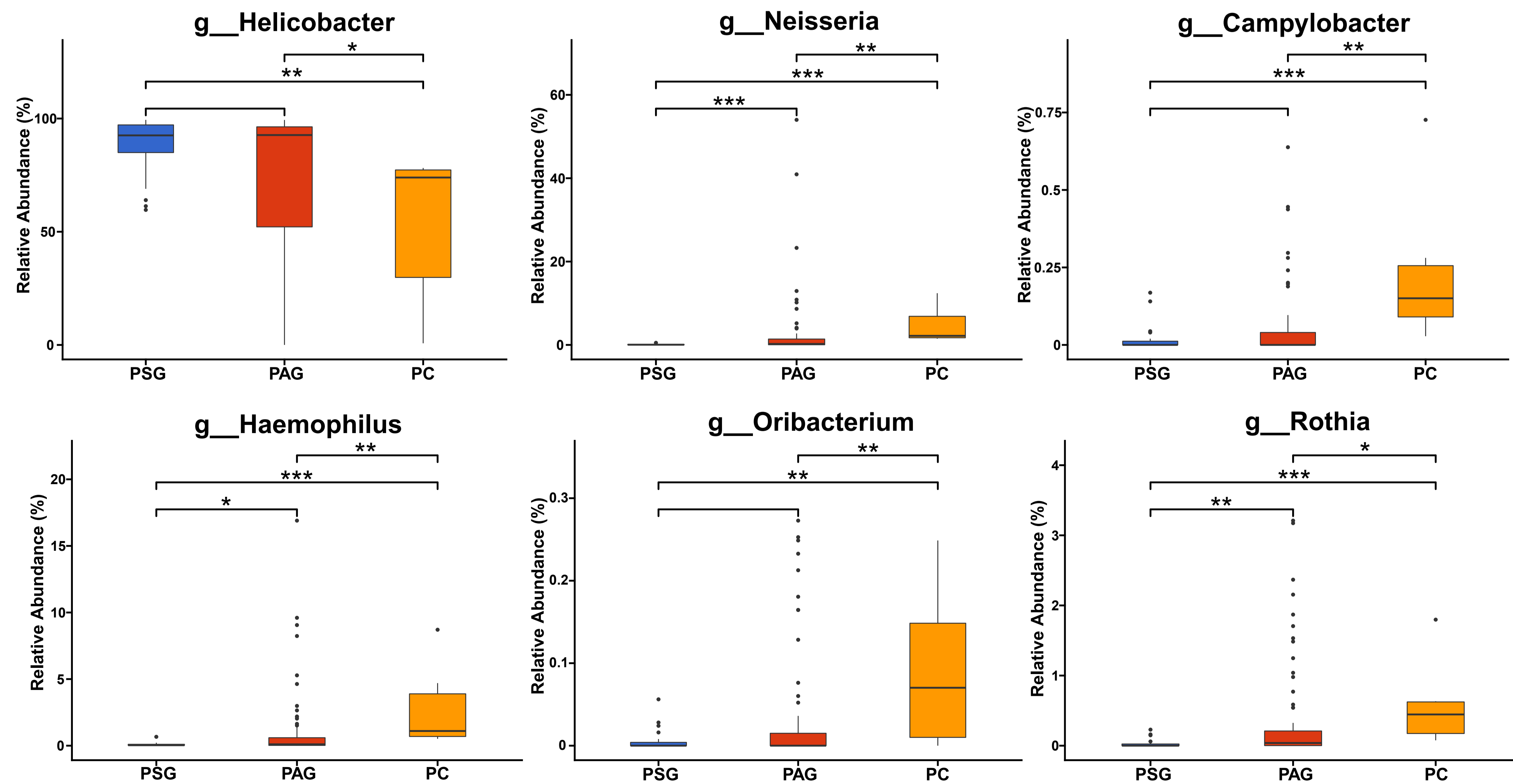

Supplement: Supplementary file 4 — Supplementary Material 4 [file 13402_2024_965_MOESM4_ESM.pdf]

**a**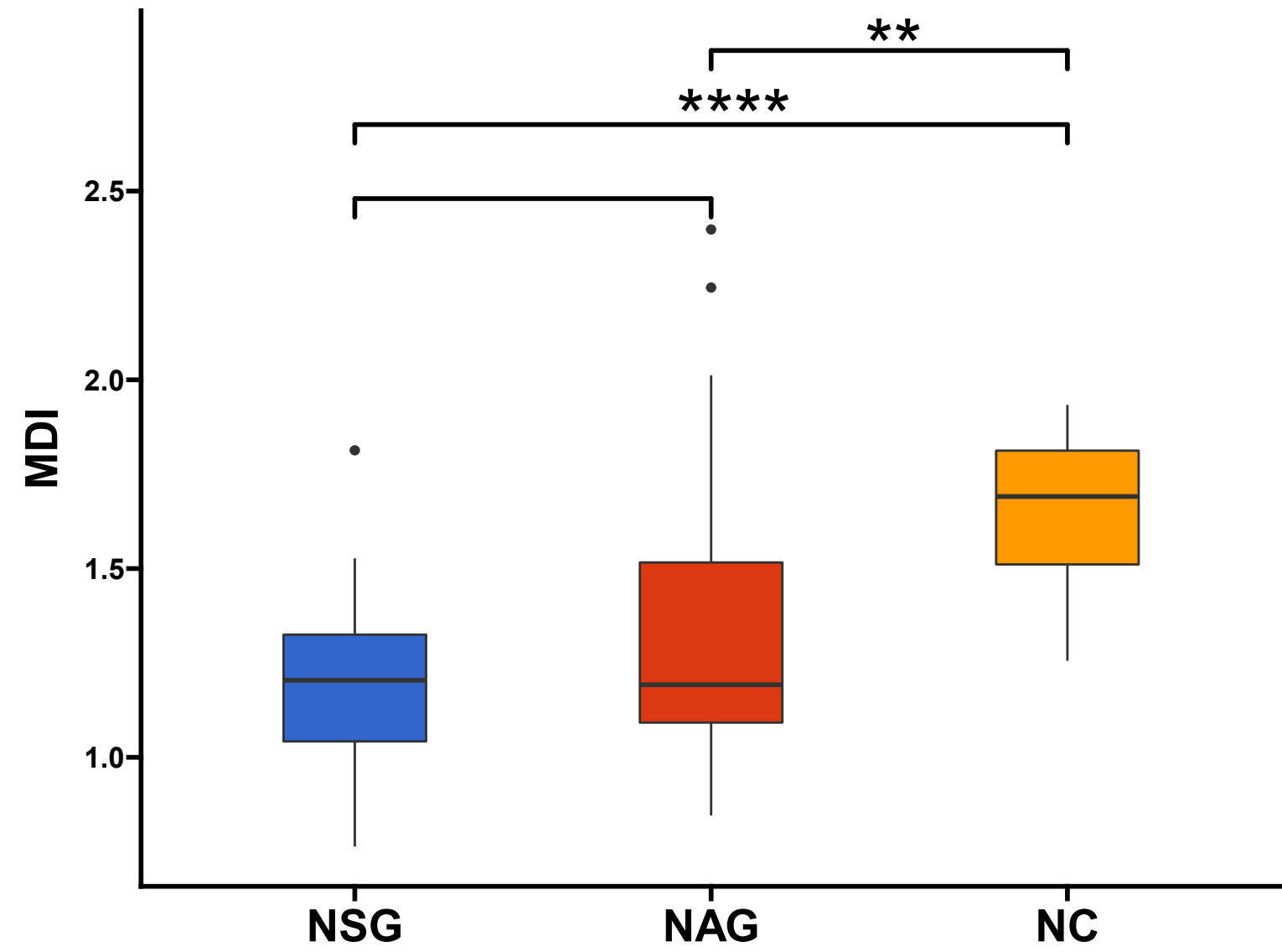**b**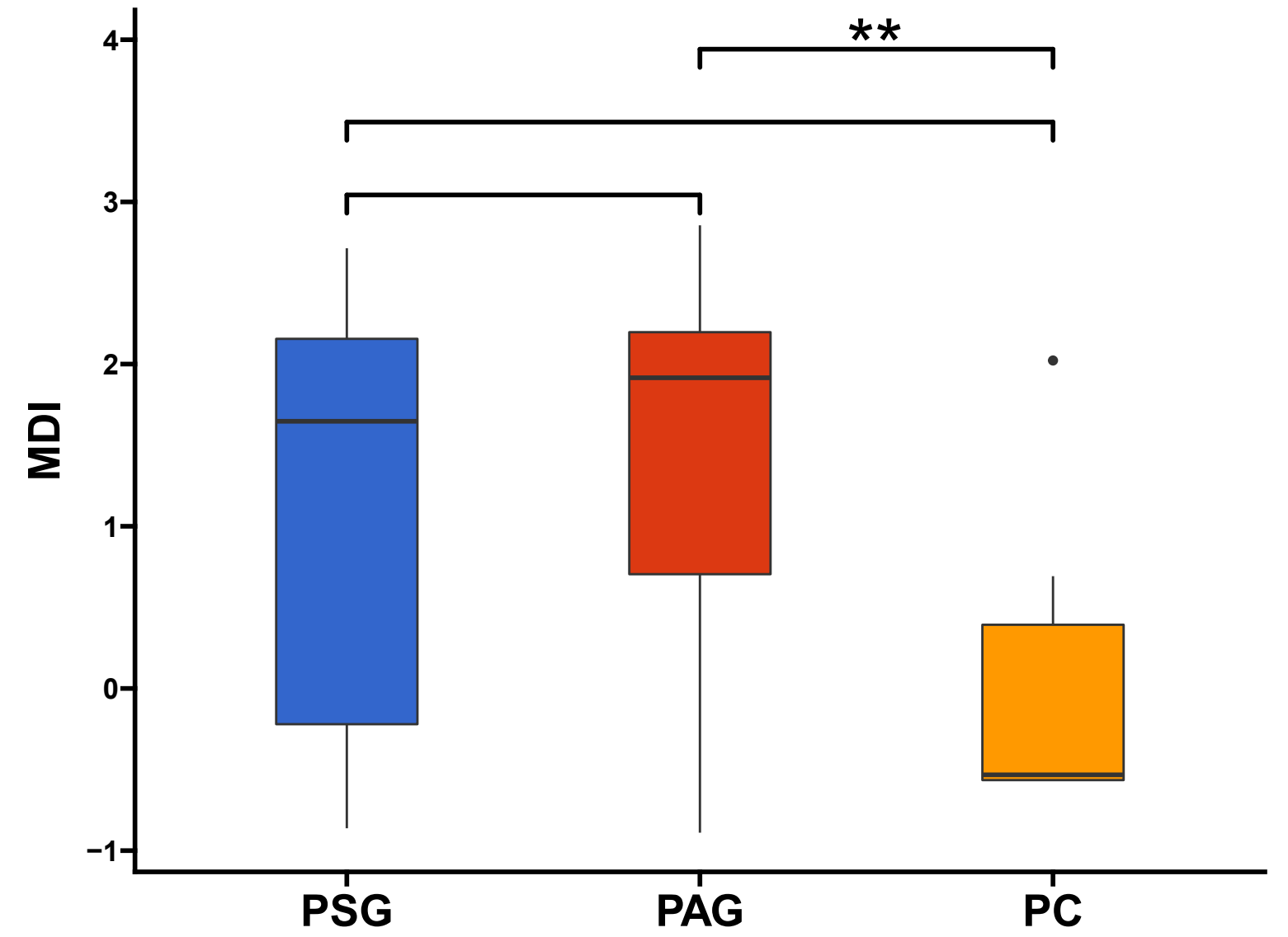

Supplement: Supplementary file 5 — Supplementary Material 5 [file 13402_2024_965_MOESM5_ESM.pdf]

a

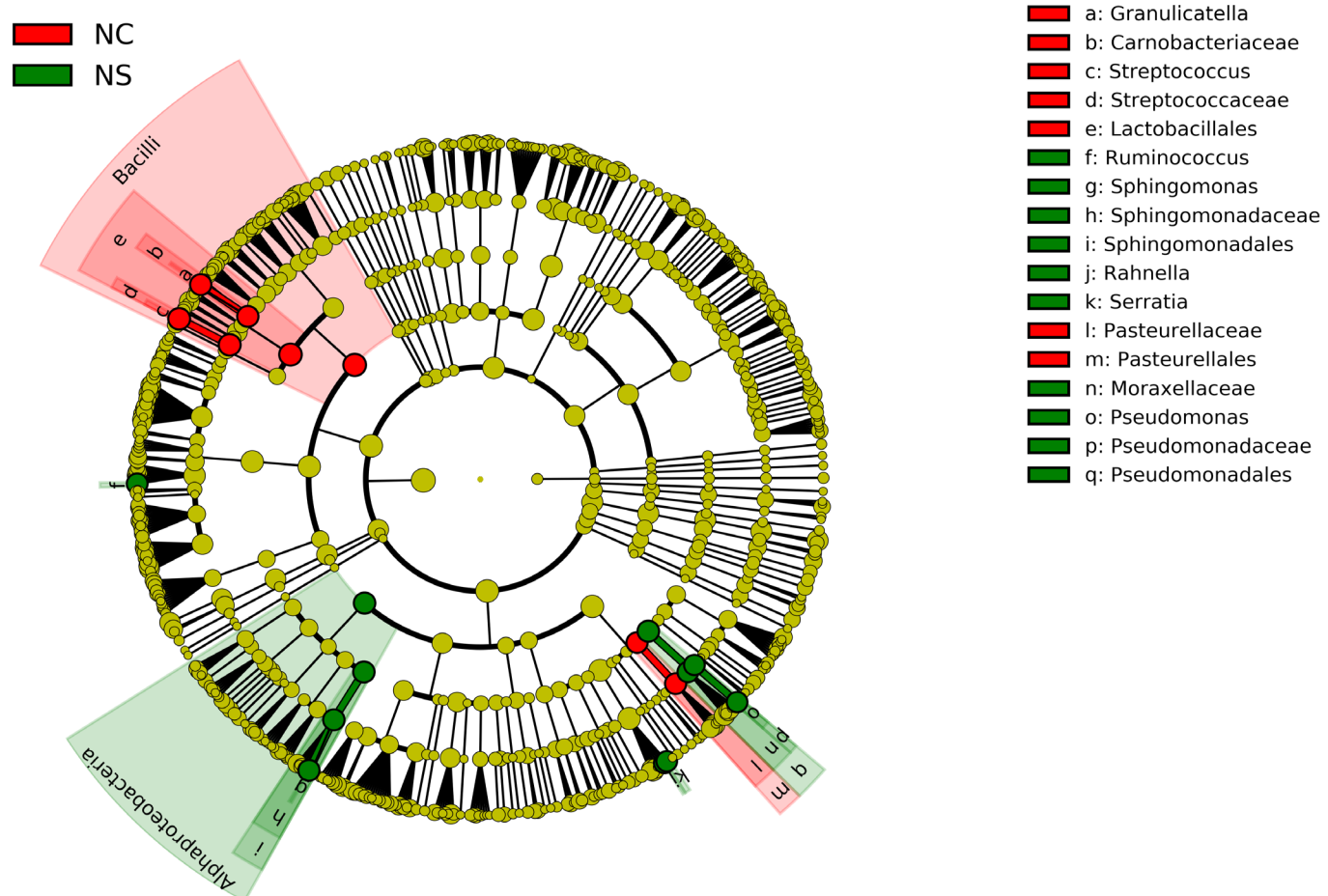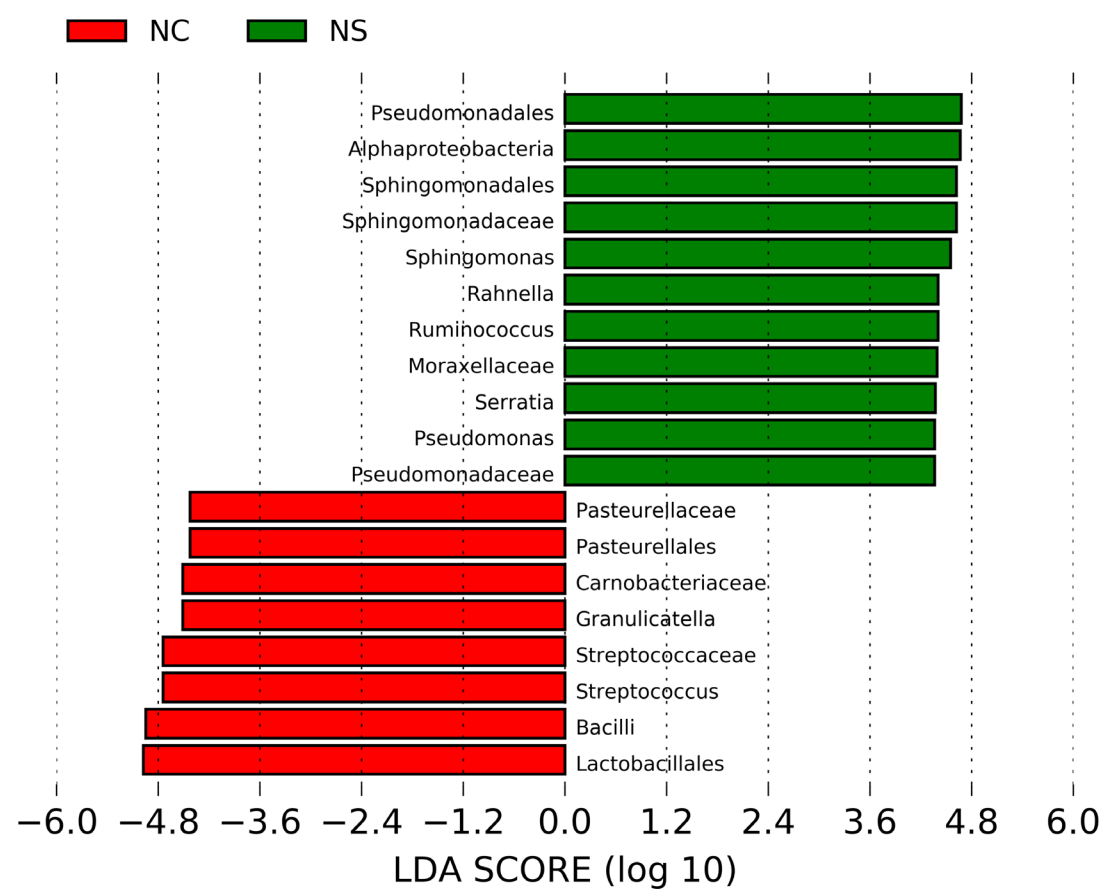

b

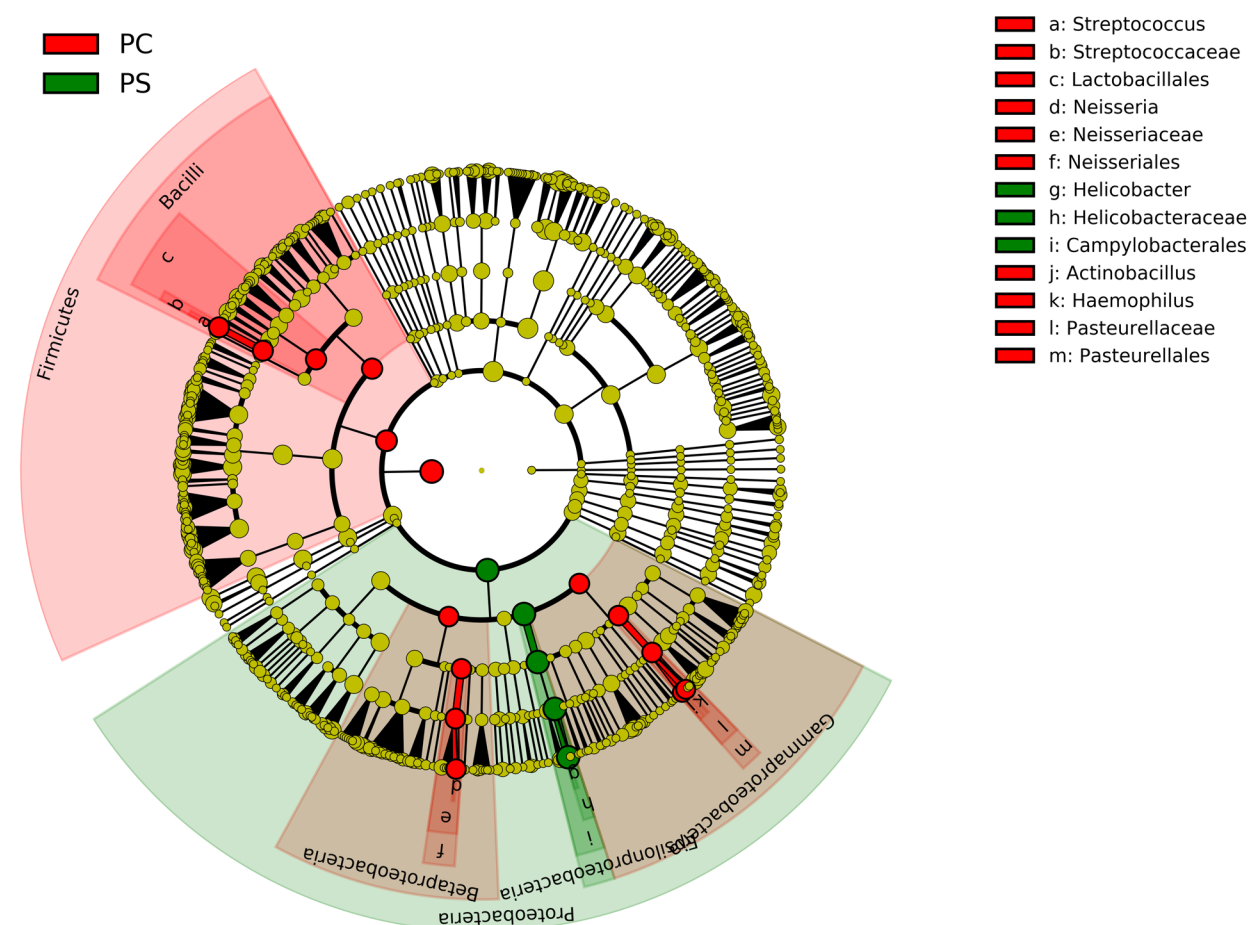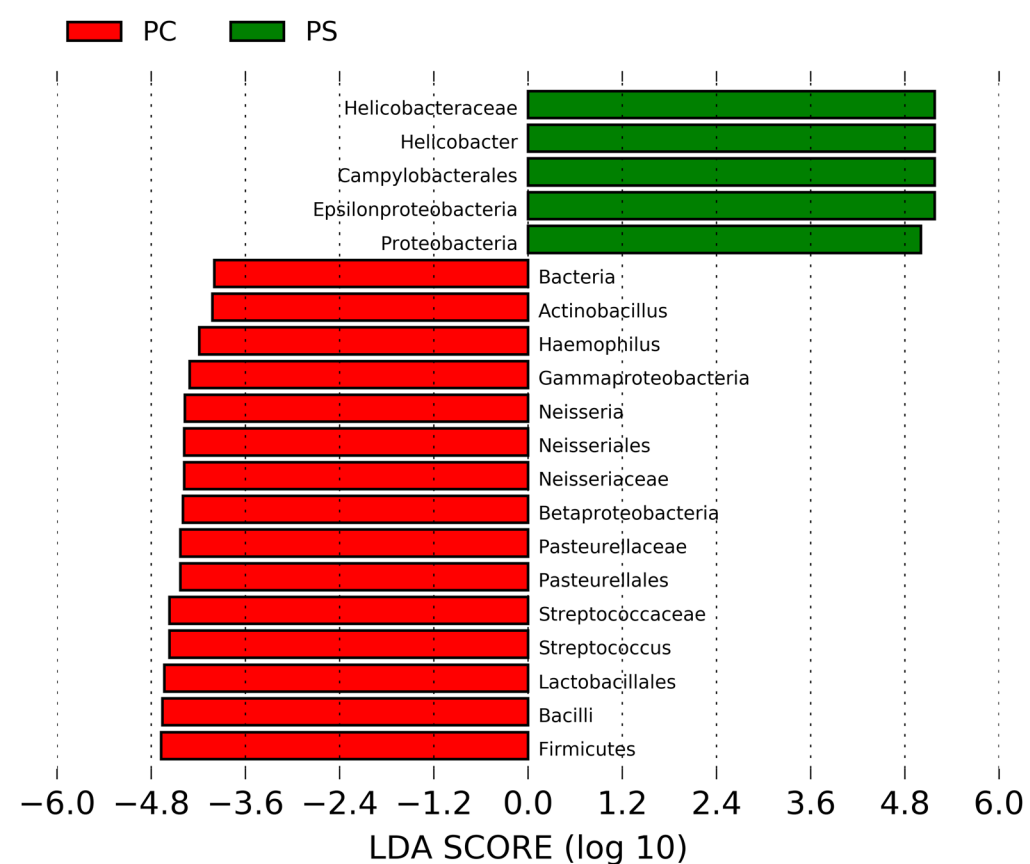

Supplement: Supplementary file 6 — Supplementary Material 6 [file 13402_2024_965_MOESM6_ESM.pdf]

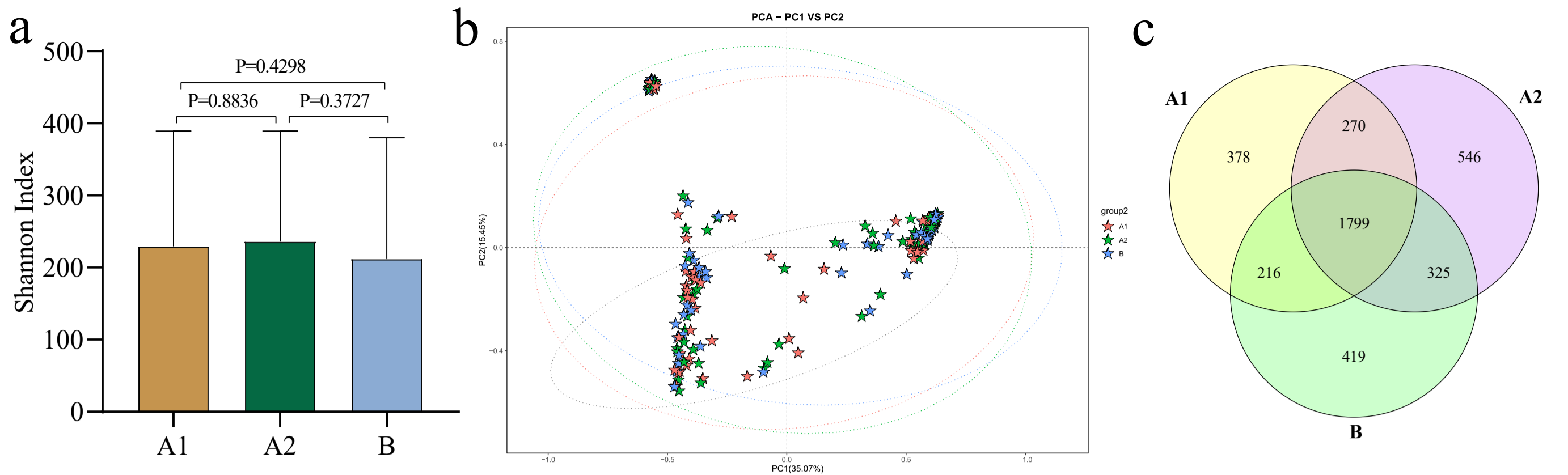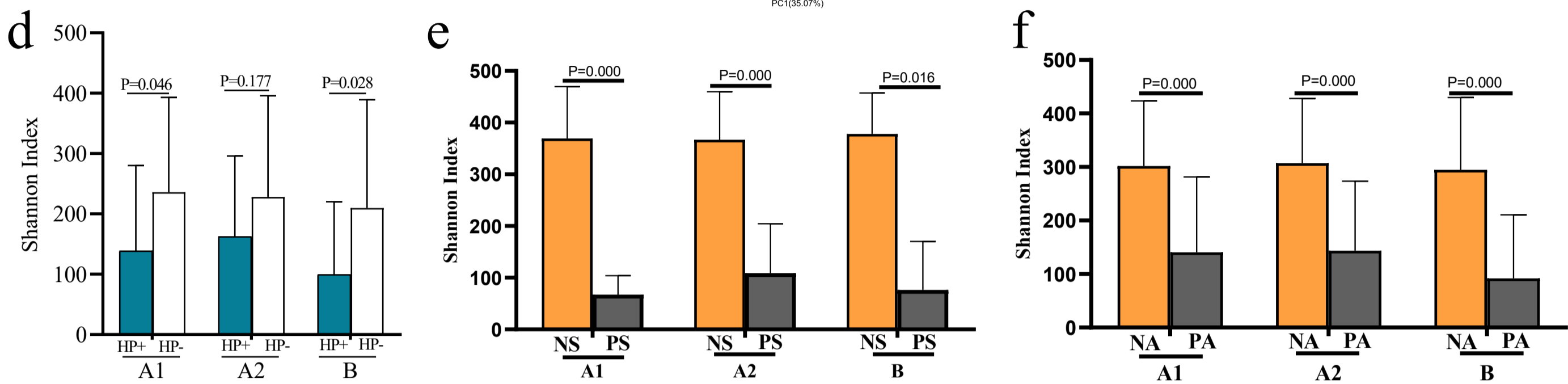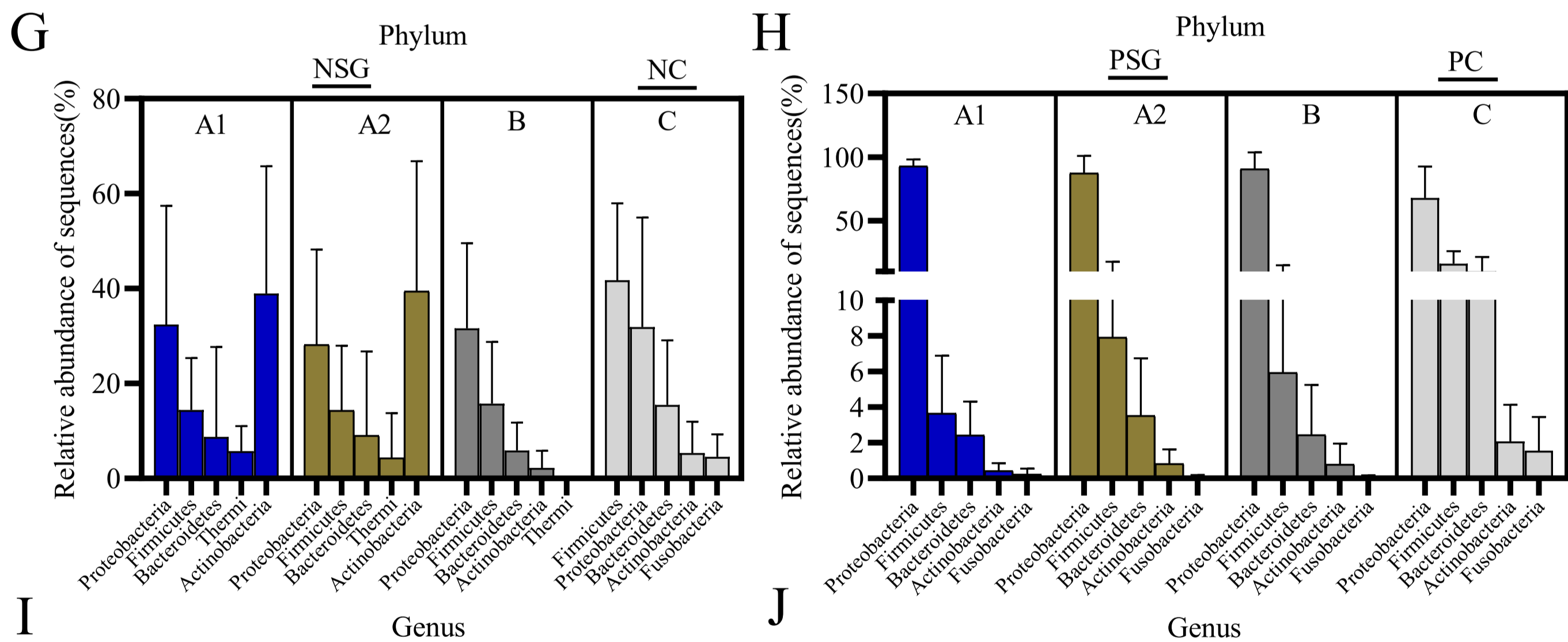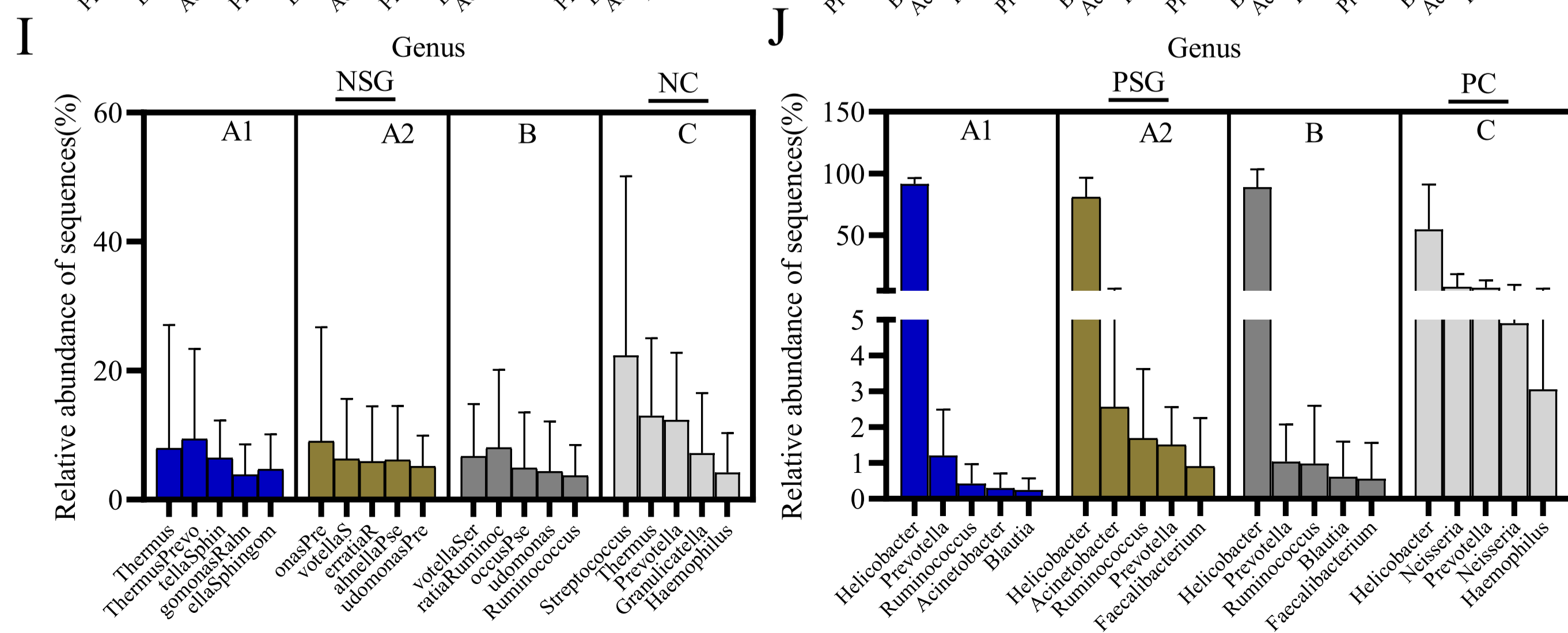

Supplement: Supplementary file 7 — Supplementary Material 7 [file 13402_2024_965_MOESM7_ESM.pdf]
