# Supplementary material for: Comprehensive multiomics analysis of the signatures of gastric mucosal bacteria and plasma metabolites across different stomach microhabitats in the development of gastric cancer
Source: Cell Oncol (Dordr). 2024 Jul 4;48(1):139–59. doi: 10.1007/s13402-024-00965-3 (PMC11850404; doi:10.1007/s13402-024-00965-3)
Supplement: Supplementary file 8 — Supplementary Material 8 [file 13402_2024_965_MOESM8_ESM.pdf]

COG gene function categories

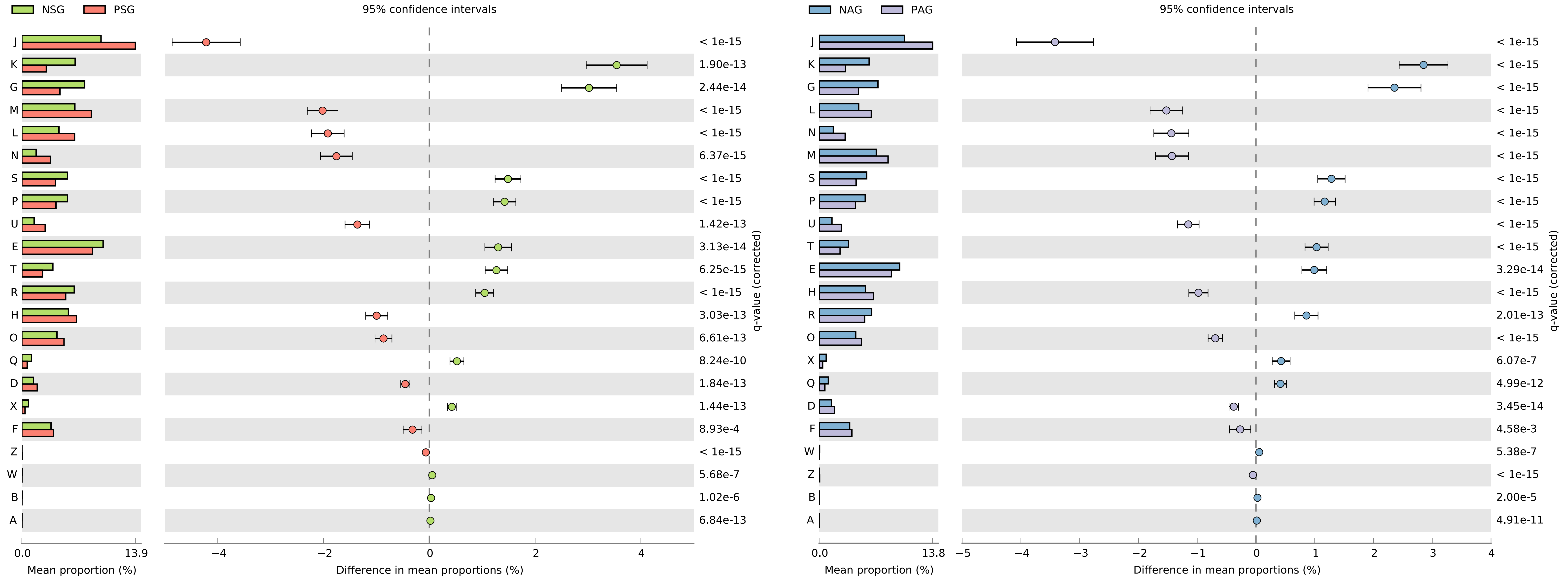

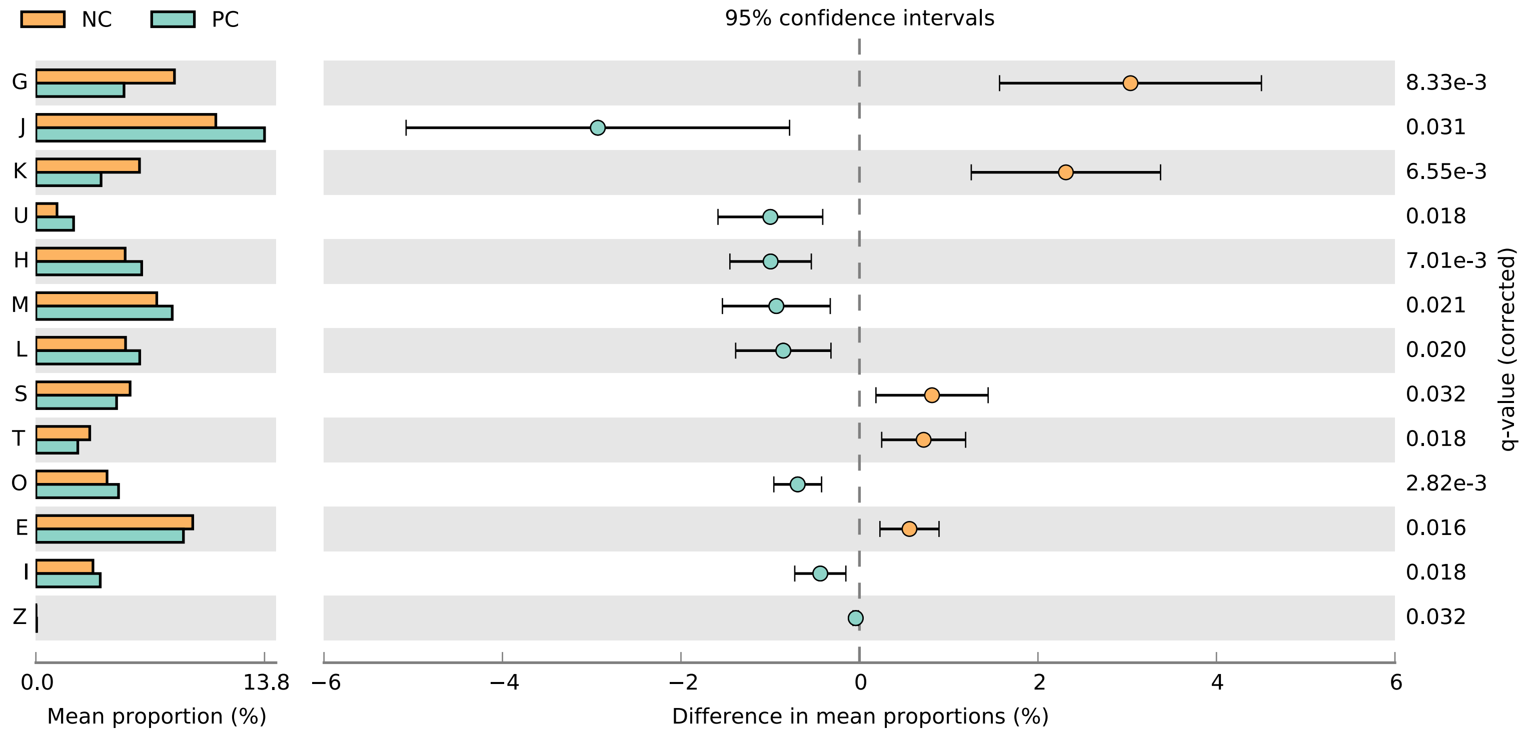

Categories

A: RNA processing and modification
B: Chromatin structure and dynamics
C: Energy production and conversion
D: Cell cycle control, cell division, chromosome partitioning
E: Amino acid transport and metabolism
F: Nucleotide transport and metabolism
G: Carbohydrate transport and metabolism
H: Coenzyme transport and metabolism
I: Lipid transport and metabolism
J: Translation, ribosomal structure and biogenesis
K: Transcription
L: Replication, recombination and repair
M: Cell wall/membrane/envelope biogenesis
N: Cell motility
O: Posttranslational modification, protein turnover, chaperones
P: Inorganic ion transport and metabolism
Q: Secondary metabolites biosynthesis, transport and catabolism
R: General function prediction only
S: Function unknown
T: Signal transduction mechanisms
U: Intracellular trafficking, secretion, and vesicular transport
V: Defense mechanisms
W: Extracellular structures
X: Mobilome: prophages, transposons
Z: Cytoskeleton
