# Supplementary material for: Comprehensive multiomics analysis of the signatures of gastric mucosal bacteria and plasma metabolites across different stomach microhabitats in the development of gastric cancer
Source: Cell Oncol (Dordr). 2024 Jul 4;48(1):139–59. doi: 10.1007/s13402-024-00965-3 (PMC11850404; doi:10.1007/s13402-024-00965-3)
Supplement: Supplementary file 9 — Supplementary Material 9 [file 13402_2024_965_MOESM9_ESM.pdf]

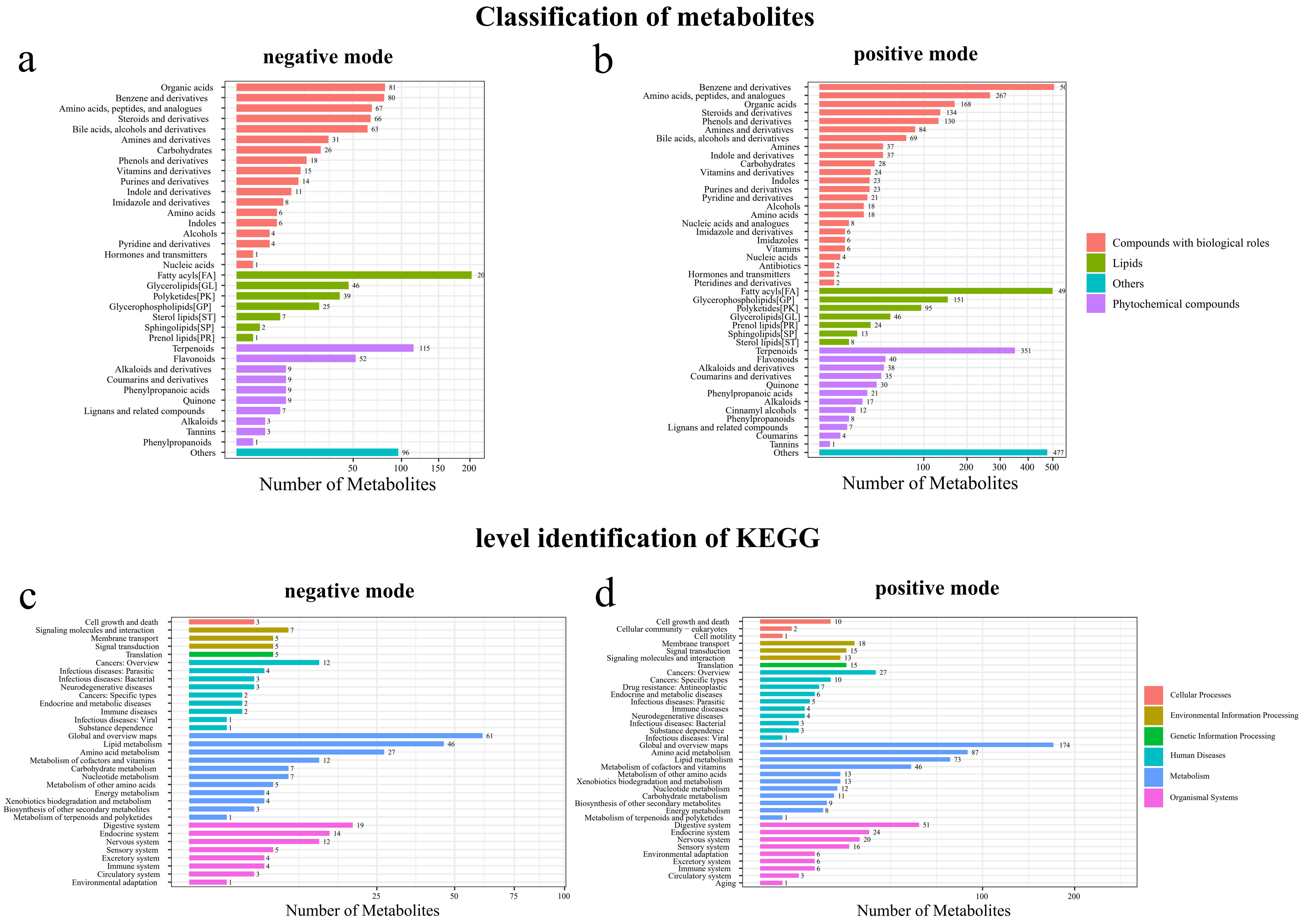
Classification of metabolites
a
negative mode
Organic acids 81
Benzene and derivatives 80
Amino acids, peptides, and analogues 67
Steroids and derivatives 66
Bile acids, alcohols and derivatives 63
Amines and derivatives 31
Carbohydrates 26
Phenols and derivatives 18
Vitamins and derivatives 15
Purines and derivatives 14
Indole and derivatives 11
Imidazole and derivatives 8
Amino acids 6
Indoles 6
Alcohols 4
Pyridine and derivatives 4
Hormones and transmitters 1
Nucleic acids 1
Fatty acyls[FA] 20
Glycerolipids[GL] 46
Polyketides[PK] 39
Glycerophospholipids[GP] 25
Sterol lipids[ST] 7
Sphingolipids[SP] 2
Prenol lipids[PR] 1
Terpenoids 115
Flavonoids 52
Alkaloids and derivatives 9
Coumarins and derivatives 9
Phenylpropanoic acids 9
Quinone 9
Lignans and related compounds 7
Alkaloids 3
Tannins 3
Phenylpropanoids 1
Others 96
50 100 150 200
Number of Metabolites
b
positive mode
Benzene and derivatives 50
Amino acids, peptides, and analogues 267
Organic acids 168
Steroids and derivatives 134
Phenols and derivatives 130
Amines and derivatives 84
Bile acids, alcohols and derivatives 69
Amines 37
Indole and derivatives 37
Carbohydrates 28
Vitamins and derivatives 24
Indoles 23
Purines and derivatives 23
Pyridine and derivatives 21
Alcohols 18
Amino acids 18
Nucleic acids and analogues 8
Imidazole and derivatives 6
Imidazoles 6
Vitamins 6
Nucleic acids 4
Antibiotics 2
Hormones and transmitters 2
Pteridines and derivatives 2
Fatty acyls[FA] 49
Glycerophospholipids[GP] 151
Polyketides[PK] 95
Glycerolipids[GL] 46
Prenol lipids[PR] 24
Sphingolipids[SP] 13
Sterol lipids[ST] 8
Terpenoids 351
Flavonoids 40
Alkaloids and derivatives 38
Coumarins and derivatives 35
Quinone 30
Phenylpropanoic acids 21
Alkaloids 17
Cinnamyl alcohols 12
Phenylpropanoids 8
Lignans and related compounds 7
Coumarins 4
Tannins 1
Others 477
100 200 300 400 500
Number of Metabolites
Compounds with biological roles
Lipids
Others
Phytochemical compounds
level identification of KEGG
c
negative mode
Cell growth and death 3
Signaling molecules and interaction 7
Membrane transport 5
Signal transduction 5
Translation 5
Cancers: Overview 12
Infectious diseases: Parasitic 4
Infectious diseases: Bacterial 3
Neurodegenerative diseases 3
Cancers: Specific types 2
Endocrine and metabolic diseases 2
Immune diseases 2
Infectious diseases: Viral 1
Substance dependence 1
Global and overview maps 61
Lipid metabolism 46
Amino acid metabolism 27
Metabolism of cofactors and vitamins 12
Carbohydrate metabolism 7
Nucleotide metabolism 7
Metabolism of other amino acids 5
Energy metabolism 4
Xenobiotics biodegradation and metabolism 4
Biosynthesis of other secondary metabolites 3
Metabolism of terpenoids and polyketides 1
Digestive system 19
Endocrine system 14
Nervous system 12
Sensory system 5
Excretory system 4
Immune system 4
Circulatory system 3
Environmental adaptation 1
25 50 75 100
Number of Metabolites
d
positive mode
Cell growth and death 10
Cellular community − eukaryotes 2
Cell motility 1
Membrane transport 18
Signal transduction 15
Signaling molecules and interaction 13
Translation 15
Cancers: Overview 27
Cancers: Specific types 10
Drug resistance: Antineoplastic 7
Endocrine and metabolic diseases 6
Infectious diseases: Parasitic 5
Immune diseases 4
Neurodegenerative diseases 4
Infectious diseases: Bacterial 3
Substance dependence 3
Infectious diseases: Viral 1
Global and overview maps 174
Amino acid metabolism 87
Lipid metabolism 73
Metabolism of cofactors and vitamins 46
Metabolism of other amino acids 13
Xenobiotics biodegradation and metabolism 13
Nucleotide metabolism 12
Carbohydrate metabolism 11
Biosynthesis of other secondary metabolites 9
Energy metabolism 8
Metabolism of terpenoids and polyketides 1
Digestive system 51
Endocrine system 24
Nervous system 20
Sensory system 16
Environmental adaptation 6
Excretory system 6
Immune system 6
Circulatory system 3
Aging 1
100 200
Number of Metabolites
Cellular Processes
Environmental Information Processing
Genetic Information Processing
Human Diseases
Metabolism
Organismal Systems
